# Fast Growth of Centimeter-Scale Molybdenum Disulfide Single Crystal for Energy-Efficient Logic Circuits

**DOI:** 10.34133/research.1117

**Published:** 2026-01-28

**Authors:** Biyuan Zheng, Hui Wang, Yizhe Wang, Weihao Zheng, Yong Liu, Guangcheng Wu, Miaomiao Li, Sha Wang, Xingxia Sun, Chenguang Zhu, Xin Yang, Zheyuan Xu, Mengjian Zhu, Li Xiang, Dong Li, Anlian Pan

**Affiliations:** ^1^ Hunan Institute of Optoelectronic Integration, State Key Laboratory of Advanced Design and Manufacturing Technology for Vehicle, College of Materials Science and Engineering, Hunan University, Changsha 410082, China.; ^2^ College of Advanced Interdisciplinary Studies & Hunan Provincial Key Laboratory of Novel Nano-Optoelectronic Information Materials and Devices, National University of Defense Technology, Changsha 410073, China.; ^3^ School of Physics and Electronics, Hunan Normal University, Changsha 410081, China.

## Abstract

Two-dimensional transition metal dichalcogenides (TMDs) are promising candidates for next-generation electronics, but their future application is hindered by the inherently slow growth kinetics of conventional vapor deposition methods, particularly for the synthesis of large-area single-crystal films. Here, we demonstrate a source-confined chemical vapor deposition strategy that enables the fast synthesis of centimeter-scale MoS_2_ single-crystal films within just 10 min. An optimized sandwich-structured Mo source was employed to ensure a concentration-balanced metal supply under sodium chloride catalysis, followed by sulfurization to form MoS_2_. The films nucleate uniformly and directionally on the miscut C/A sapphire substrate positioned 2 cm upstream of the Mo source, achieving high crystal quality with a low sulfur vacancy density of 8.49 × 10^12^ cm^−2^. Additionally, these films support the development of high-performance enhancement-mode MoS_2_ field-effect transistors, exhibiting excellent transport performances, including a high on–off ratio of 10^8^, an average positive threshold voltage of 1.71 ± 0.32 V, an average mobility of 34.28 ± 0.46 cm^2^ V^−1^ s^−1^, and an average subthreshold swing of 155.8 ± 33.7 mV dec^−1^. Furthermore, high-performance rail-to-rail inverter gates and logic circuits with low power consumption (<0.3 nW) were successfully demonstrated, underscoring the potential of these MoS_2_ films for integrated circuit applications. This work offers a scalable and reliable approach for the fast growth of large-scale TMDs single-crystal films, accelerating their future applications in next-generation electronics.

## Introduction

The scaling of silicon integrated circuits (ICs) is gradually approaching its limit due to the drain-induced barrier lowering, mobility degradation, high off-state currents, and power consumption increase caused by the short-channel effect. Atomically thin 2-dimensional (2D) transition metal dichalcogenides (TMDs) semiconductors, such as MoS_2_, featuring dangling-bond-free surfaces, excellent mechanical properties, flexibility, and outstanding optoelectronic properties, are regarded as promising channel materials for future ICs [1–7]. Drawing on the lessons from the development of silicon-based circuits, achieving fast growth of high-quality MoS_2_ single-crystal films is crucial for realizing its practical applications. However, achieving fast growth of TMDs single-crystal films remains an important technological challenge.

Extensive research efforts have culminated in the successful fabrication of triangular MoS_2_ nanosheets and continuous films using numerous methods [8–12]. The chemical vapor deposition (CVD) method exhibits distinct advantages for synthesizing high-quality MoS_2_ single-crystal films, making it a highly promising candidate for practical applications. Recently, considerable progress has been achieved in the preparation of MoS_2_ via low-pressure CVD (LPCVD), including the successful synthesis of wafer-scale single-crystal films through the merging of multiple single-oriented MoS_2_ domains [11–19]. However, several challenges remain unresolved in this field. For instance, achieving fast growth is difficult. In current LPCVD processes for synthesizing inch-scale MoS_2_ single-crystal films, the large separation distance (>30 cm) between the metal precursor source and the substrate leads to inadequate reactant concentration. This results in an extended synthesis time of 30 to 60 min and low growth rates of less than 0.5 cm^2^ min^−1^ [11,12,15,16,19]. A stable, efficient, and well-balanced precursor supply is essential for substantially reducing the growth time, which is a critical factor in enabling the fast growth of MoS_2_ films. Therefore, optimizing the growth process and minimizing the synthesis duration are crucial for the production and practical deployment of large-area MoS_2_ single-crystal films. Furthermore, field-effect transistors (FETs) made from wafer-scale LPCVD-grown MoS_2_ films typically exhibit depletion-mode operation (D-mode, indicated by a large negative threshold voltage [*V*_TH_] and drain current at zero gate voltage), a direct consequence of their intrinsic n-type doping [11,13,15,16]. The utilization of such D-mode devices requires the integration of either additional negative bias circuitry or complex compensation circuits to ensure proper switching operation. Consequently, the development of enhancement-mode (E-mode) FETs based on high-quality MoS_2_ is of critical importance, as they enable the construction of rail-to-rail inverter gates with superior noise margins and ultralow power consumption.

In this work, we developed a metal source-confined Mo source (Mo foil/MoO_3_ + NaCl/Mo foil) and strategically positioned it downstream of the sapphire substrate to enable the fast growth of the high-quality MoS_2_ single-crystal films. The intentionally designed sandwiched Mo source ensured a continuous, stable, and efficient supply of gas-phase Mo at high growth temperatures, preventing the formation of multilayer and randomly oriented MoS_2_ domains. As a result, we successfully synthesized centimeter-scale MoS_2_ single-crystal film in just 10 min. High-resolution scanning transmission electron microscopy (HRSTEM) characterization revealed a sulfur vacancy density of about 8.49 × 10^12^ cm^−2^, confirming the high quality of the achieved MoS_2_ films. The as-grown MoS_2_ single-crystal film was further transferred onto a 30-nm Al_2_O_3_ dielectric, and independent back-gate controlled FETs were fabricated. These FETs presented a high on–off ratio, positive *V*_TH_, high mobility, and small subthreshold swing (SS). Based on the high-performance E-mode FETs, functional logic circuits, including inverters, NAND, NOR, and AND gates, were fabricated, all with low power consumption, demonstrating their potential for low-power ICs applications.

## Results

**Fig. 1.**
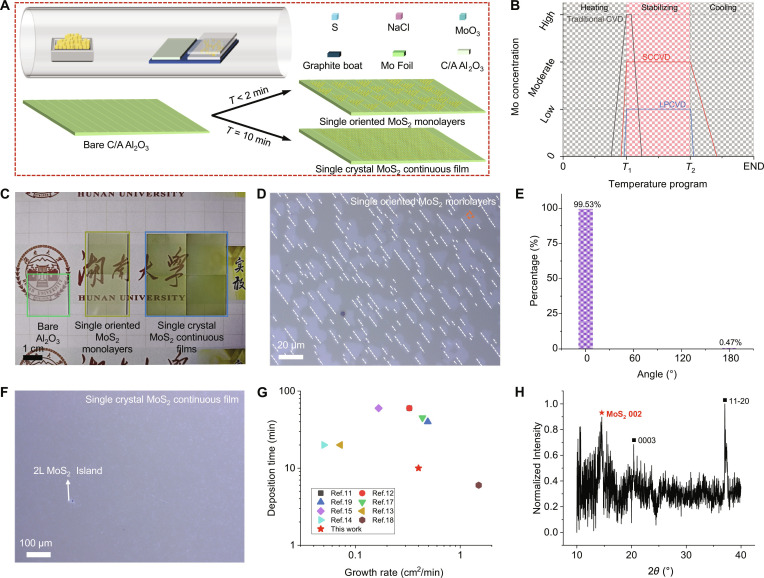
Fast growth of centimeter-scale MoS_2_ single-crystal films. (A) Schematic illustration of SCCVD growth of single-oriented MoS_2_ monolayers or MoS_2_ single-crystal film. (B) Comparison of the Mo source concentration in different synthesis methods. (C) Photographs of bare C/A sapphire substrate (highlighted by the green box, 1 piece), single-oriented MoS_2_ monolayers on sapphire substrates (highlighted by the yellow box, 2 pieces), and MoS_2_ single-crystal films on sapphire substrates (highlighted by the blue box, 4 pieces). Scale bar, 1 cm. (D) Optical image of single-oriented MoS_2_ monolayers; the white dotted lines highlight the aligned orientation. (E) Statistical distribution of MoS_2_ domains aligned with and without the atomic steps. (F) Optical image of MoS_2_ single-crystal film. (G) Deposition time and growth rate summary of single-crystal TMDs. (H) XRD pattern of MoS_2_ single-crystal film on sapphire substrate.

The metal source-confined CVD (SCCVD) method (see more details in Materials and Methods) was employed to synthesize centimeter-scale MoS_2_ single-crystal films. Briefly, in the beginning, we fabricated an intentionally designed sandwiched Mo source, which consisted of 2 Mo foils (2 cm × 2 cm) and mixed powders containing MoO_3_ and NaCl (Fig. 1A), which were flattened between the 2 Mo foils. NaCl catalyst is helpful to synthesize MoS_2_ more effectively and avoid MoO_2_ by-products. The possible chemical reactions are as follows: 2MoO_3_ + 2NaCl⟶Na_2_MoO_4_ + MoO_2_Cl_2_ and MoO_2_Cl_2_ + 3S⟶MoS_2_ + SO_2_ + Cl_2_. A high-temperature pre-annealed miscut C/A sapphire substrate (2 cm × 2 cm) and the sandwiched Mo source were placed on a graphite sheet (2 cm × 6 cm), where the sapphire substrate was located at the upstream position, and the distance between them was 2 cm. Notably, the designed sandwiched Mo source has 3 advantages: (a) preventing premature nucleation; (b) ensuring appropriate reactant concentration; and (c) providing a continuous and uniform supply of reaction source. Based on previous reports [11,19], if the deposition temperature is lower than 850 °C, the MoS_2_ domains would be randomly arranged. In our case, evaporable MoO_3_ powder was confined between 2 Mo foils, effectively avoiding the premature nucleation of MoS_2_. Also, the slow release of the Mo can provide a more sustained supply of metal sources. In addition, the sapphire substrate was placed at the upstream position of the Mo source to avoid uneven nucleation caused by carrier gas [20]. Owing to the above design, the single-oriented MoS_2_ monolayers or MoS_2_ single-crystal films were efficiently synthesized. Notably, compared with previous reports [11,12], the growth time in our work was decreased to less than 2 min for single-oriented monolayers and 10 min for single-crystal films. As shown in Fig. 1B, we vividly compare the relationship between Mo concentration and the growth process in this work (SCCVD: red line in Fig. 1B) and previous experiments (traditional CVD: gray line in Fig. 1B; LPCVD: blue line in Fig. 1B). For the traditional CVD growth of MoS_2_ nanoflakes, the MoO_3_ or MoO_2_ as Mo source was placed at the center position, and the substrate was placed face down above the Mo source. In this case, when the temperature reaches the deposition temperature, the Mo source will evaporate rapidly, resulting in excessive Mo concentration. Hence, the LPCVD, which was now widely used for the growth of TMDs single-crystal films, was introduced, where the Mo source was placed in the upstream position, and O_2_ gas was used to promote evaporation and prevent by-products (MoO_2_ nanosheets), and the whole reaction was carried out at low pressure. However, owing to the ultralong distance between the Mo source and substrate (more than 30 to 40 cm in length) [17], the Mo concentration in LPCVD was too low, resulting in long growth time (30 to 60 min) and low growth rate (<0.5 cm^2^ min^−1^). Notably, in this work, the evaporable MoO_3_ and NaCl powders were confined by 2 Mo foils, which successfully avoided the early evaporation, the high Mo concentration, the short supply of Mo source in the traditional CVD method, and the low Mo concentration in the LPCVD method.

Figure 1C shows the photograph of the bare C/A sapphire substrate, the single-oriented MoS_2_ monolayers, and the MoS_2_ single-crystal films. It is obvious that the bare sapphire presented a translucent color, and the MoS_2_ single-crystal films showed a darker green color than single-oriented MoS_2_ monolayers due to higher coverage. Figure S1 gives the atomic force microscope (AFM) images of a pre-annealed bare sapphire substrate, demonstrating that the substrate had atomic steps. The miscut angle could be determined by the x-ray diffraction (XRD) rocking curve [11]. As shown in Fig. S2, the measured angle was 0.78°. Figure 1D shows the optical image of the as-grown MoS_2_ domains, which demonstrates that almost all the domains were aligned unidirectionally. Further statistical analysis presented that around 99.53% of the MoS_2_ domains were single-oriented (Fig. 1E). It should be noted that the MoS_2_ domains obtained in this study deviate from the conventional triangular or hexagonal shapes. This deviation may be attributed to 2 primary factors under our current growth conditions: firstly, the Mo and S precursor ratio may not yet be optimized; secondly, the growth rate is likely too high. Notably, we have also observed MoS_2_ domain morphologies resembling diamonds (Fig. S3A) and approximate triangles (Fig. S3B). Only if the deposition time was increased to 10 min could the MoS_2_ single-crystal films be obtained by merging the boundaries of these single-oriented MoS_2_ domains. Except for few 2-layer inlands, a clean, flat, and seamless MoS_2_ film is presented in Fig. 1F. More optical images of the obtained MoS_2_ films are shown in Fig. S4. Figure 1G summarizes the growth time and growth rate of single-crystal TMDs (including MoS_2_, WS_2_, and Fe-doped MoS_2_), indicating that, compared with previous papers, our deposition time is shorter, and our growth rate is faster [11–19]. Figure S5 compares the experimental results of placing the substrates at the upstream and downstream positions of the Mo source, confirming that our experimental design effectively prevented the uneven nucleation caused by the carrier gas flow. The AFM image (Fig. S6A) demonstrated that one edge of the MoS_2_ was well aligned with the atomic step of the sapphire substrate. The thickness of the MoS_2_ was measured as 0.8 nm (Fig. S6C), indicating the single-layer characteristic. A locally amplified AFM image (Fig. S6B and D) proved that the obtained material had a clean surface, which preliminarily evidenced its high crystalline quality. XRD was used to determine the crystal composition and structure of the MoS_2_ film and the sapphire substrate. As shown in Fig. 1H, the diffraction peak located at 14.35° corresponded to the (002) plane of the MoS_2_ film (JCPDS #37-1492). The diffraction peaks at 20.53°, 37.50°, and 41.7° (Fig. S7) consisted of (0003), (11–20), and (0006) planes of sapphire (JCPDS #46-1212), respectively.

**Fig. 2.**
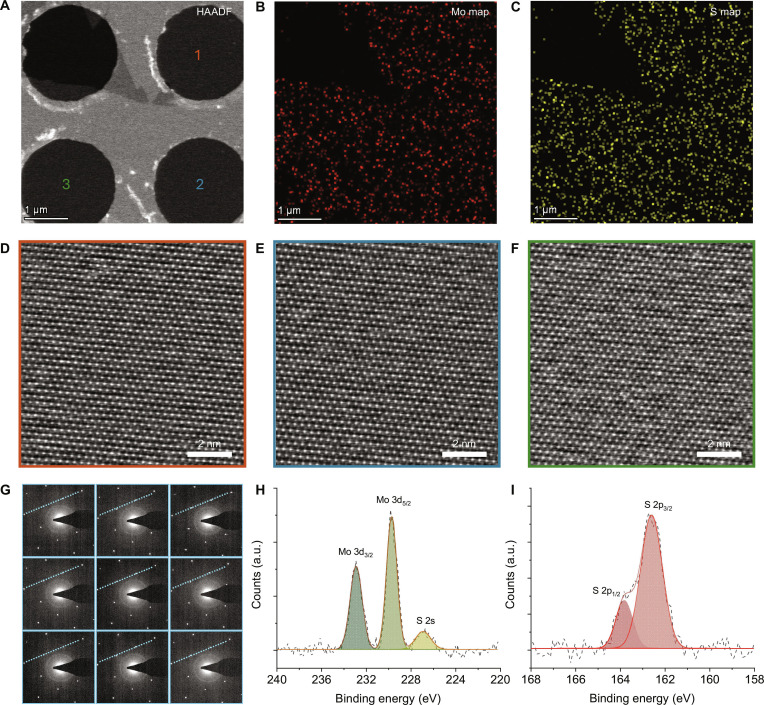
STEM and XPS characterizations of single-oriented MoS_2_ monolayers. (A) Low-magnification HAADF STEM image of 2 MoS_2_ domains with one edge merged. (B and C) The corresponding Mo and S elements’ distribution profiles. (D to F). HRSTEM images of MoS_2_ taken from points 1 to 3 in (A). (G) SAED patterns were collected from 9 random MoS_2_ domains. (H and I) XPS spectra of Mo 3d and S 2s peaks.

The chemical composition, element distribution, atomic structure, and chemical valence of the single-oriented MoS_2_ monolayers were further investigated. As shown in Figs. S8 and S9, using a 2-step wet etching transfer method, we successfully transferred our MoS_2_ domains from sapphire to copper grid for STEM characterizations (see more details in Materials and Methods). Figure 2A shows the low-magnification high-angle annular dark field (HAADF) STEM image of 2 merged MoS_2_ domains transferred from sapphire to a copper grid with ultrathin carbon film. The single STEM energy-dispersive spectroscopy (EDS) spectra (Fig. S10) and element distribution mapping image (Mo: red dots in Fig. 2B, and S: yellow dots in Fig. 2C) determined the element composition and spatial distribution of the detected samples, confirming that the samples were MoS_2_. The merging behavior of the 2 MoS_2_ domains was further studied. HRSTEM images (Fig. 2D to F) demonstrate that the 2 MoS_2_ domains are seamlessly stitched together. To quantitatively evaluate the sulfur vacancy density of the MoS_2_ film, the samples were transferred onto a copper grid with quantifoil holey carbon (Fig. S11, without ultrathin carbon film), which could present a clear atomic structure. Figure S12A gives the HRSTEM result, and the density of sulfur vacancies was calculated as 8.49 × 10^12^ cm^−2^. Figure S12B shows the comparison results of the sulfur vacancy density in this work with previous papers [6,11,17,21–26], indicating a low defect density of the MoS_2_ sample. The selected area electron diffraction (SAED) patterns collected from 9 different positions of the copper grid loaded with MoS_2_ domains are shown in Fig. 2G. All 9 patterns presented hexagonal diffraction spots with the same crystallographic orientation (highlighted with dotted blue lines in Fig. 2G), proving that our MoS_2_ domains were single-oriented. Figure S13 is the corresponding line scan profiles along the dotted blue lines in Fig. 2G, further confirming the above claims. Low-energy electron diffraction (LEED) measurements were carried out at 6 distinct locations across a continuous thin-film sample (Fig. S14). The observed diffraction patterns exhibit sharp and well-defined spots, which are nearly identical in position and intensity across all measured regions. This consistency strongly indicates the single-crystal character of the film within the spliced area, confirming its structural coherence and high crystalline quality (Fig. S15). The chemical valence of the MoS_2_ single-crystal films was investigated by x-ray photoelectron spectroscopy (XPS). Figure S16 shows the XPS full spectrum of the tested sample, in which, except for the Al 2s, O 1s, Na 1s, and C 1s peaks from the substrate, strong Mo 3d and S 2p peaks were obtained. As shown in Fig. 2H, the binding energy peaks located at 232.9 (Mo 3d_3/2_) and 229.7 eV (Mo 3d_5/2_) resulted from the Mo oxidation state of +4. The binding energy peak at 226.9 eV (S 2s), along with the S 2p_1/2_ (163.8 eV) and S 2p_3/2_ (162.6 eV) peaks, demonstrated the existence of S^2−^, which was consistent with previous reports of MoS_2_ films (Fig. 2I) [27–29]. The Mo/S ratio was finally calculated as 1:1.8 (Table S1), confirming the formation of the MoS_2_ film.

**Fig. 3.**
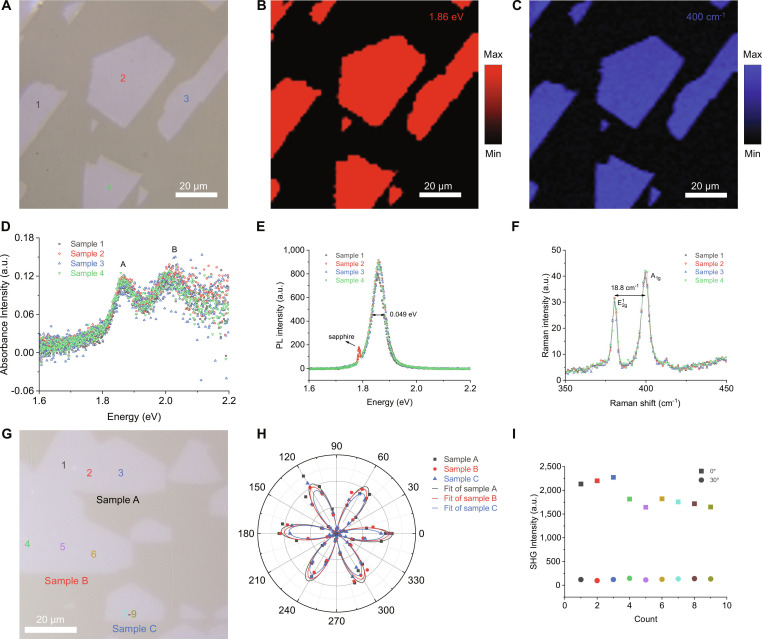
Absorption, PL, Raman, and SHG characterizations of single-oriented MoS_2_ monolayers. (A) Optical image of the MoS_2_ samples for absorption, PL, and Raman characterizations. (B and C) Wavelength-selected PL intensity mapping (B) and Raman shift-selected Raman intensity mapping (C) images of the single-oriented MoS_2_ monolayers. (D to F) Absorption (D), PL (E), and Raman (F) spectra obtained from samples 1 to 4 in (A). (G) Optical image of MoS_2_ samples for SHG test. (H) Polar plot of the SHG intensities as a function of parallel-polarized laser angles. (I) SHG intensities of the detected samples with parallel-polarized laser angles at 0° and 30°, respectively.

The optical properties of the MoS_2_ single-oriented monolayers were investigated by absorption, photoluminescence (PL), Raman, and second harmonic generation (SHG) characterizations. Figure 3A shows the optical image of the samples to be measured, in which 4 separate MoS_2_ domains (samples 1 to 4) were selected. Figure 3D is the absorption spectra obtained from samples 1 to 4, in which 2 obvious peaks located at 1.87 and 2.02 eV were observed, corresponding to the A and B excitons of MoS_2_ monolayers, respectively. Figure 3E presents the PL spectra of these MoS_2_ monolayers. A strong PL peak located at 1.86 eV corresponded to the recombination of A excitons, and the weak peak located at 1.79 eV was from the sapphire substrate [30]. The full width at half-maximum of these spectra was as low as 0.049 eV, demonstrating the high crystal quality of our MoS_2_. For the Raman spectra (Fig. 3F), the E^1^_2g_ (380.8 cm^−1^, in-plane vibrational mode) and A_1g_ (399.6 cm^−1^, out-of-plane vibrational mode) peaks were collected. The wavenumber difference between A_1g_ and E^1^_2g_ is about 18.8 cm^−1^. The above results agreed well with previously reported MoS_2_ monolayers [6,18]. Then, the PL and Raman mapping images were measured to investigate the uniformity of the MoS_2_ domains. As shown in Fig. 3B, the wavelength-selected PL intensity mapping image demonstrated the uniform PL emission at 1.86 eV. Figure 3C gives the Raman mapping image (400 cm^−1^), further confirming the uniform formation and high quality of our MoS_2_ monolayers. The SHG was commonly used to characterize the orientation of the MoS_2_ domains. As shown in Fig. 3G, 3 merged MoS_2_ domains were selected. According to a previous paper [31], the SHG intensity in MoS_2_ monolayers follows the rule as *I* = *I*_0_ × cos^2^(2*θ*), where θ is the azimuthal angle between the polarization of the excitation laser and the armchair direction of MoS_2_ domains. Figure 3H is the polarization angle-dependent SHG intensity for the 3 MoS_2_ domains, which proves that the tested samples have a single orientation. Figure 3I and Fig. S17 show the SHG intensities collected from more positions of the MoS_2_ monolayers, in which the SHG intensities at *θ* of 0° were higher than 1,500 counts, and those at *θ* of 30° were below 100 counts, confirming that our MoS_2_ domains were well aligned.

**Fig. 4.**
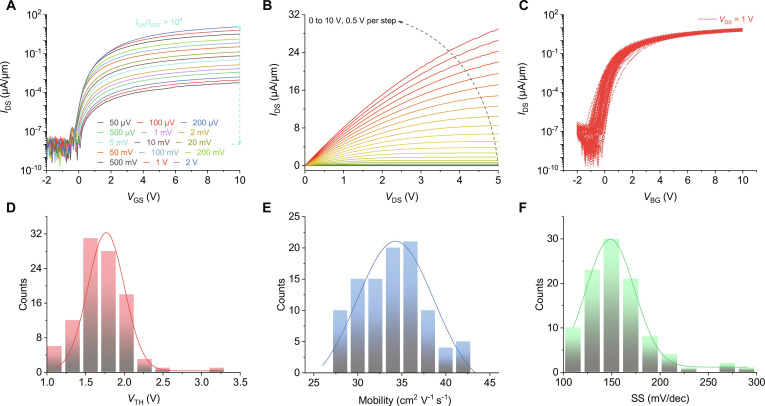
Electrical properties of the independently controlled FETs. (A) Typical transfer curves of MoS_2_ FET at *V*_DS_ from 50 μV to 2 V. (B) Output covers MoS_2_ FET at *V*_GS_ from 0 to 10 V, and 0.5 V per step. (C) Transfer covers of 100 FETs at *V*_DS_ = 1 V. (D to F) Statistical distribution of *V*_TH_ (D), mobility (E), and SS (F) of the 100 FETs.

Based on the successful growth of centimeter-scale MoS_2_ single-crystal films, the independent FETs were fabricated. The thermal release tape (TRT)-assisted wet transfer method of the MoS_2_ films and the fabrication process flow of the devices are presented in Figs. S8 and S18, respectively, in which atomic layer deposition (ALD)-processed 30 nm Al_2_O_3_ was employed as the oxide dielectric layer. The layout and optical images of the FETs are presented in Fig. S19. The 100 tested FETs were distributed over an area of 0.8 cm × 0.25 cm, with each device featuring a channel length of 10 μm and a channel width of 50 μm. Figure 4A shows the transfer curves at different drain voltages (*V*_DS_). With the *V*_DS_ increasing, the drain current (*I*_DS_) increased correspondingly, demonstrating that the device worked well both at low (50 μV) and high (2 V) drain voltage. The on–off ratio could reach up to 10^8^ at a *V*_DS_ of 2 V, proving that the MoS_2_ had excellent electrical properties. Figure 4B shows the drain current–voltage (*V*_DS_–*I*_DS_) output curves. The highest *I*_DS_ of the FET was up to 29 μA/μm at *V*_DS_ = 5 V, confirming that the grown MoS_2_ film has high quality with excellent current driving capabilities. The output curves showed linear (Fig. S20A) and saturation (Fig. S20B) current at low and high bias, respectively, exhibiting the characterization of an ohmic contact. The scalability and uniformity of the MoS_2_ film FETs were investigated, and the corresponding results are shown in Fig. 4C and Fig. S21, in which the transfer curves of 100 FETs were displayed. A statistics histogram analysis shows that the FETs exhibited high on-state current (*I*_ON_ = 6.54 ± 0.82 μA/μm), low off-state current, and high on–off ratio (*I*_ON_/*I*_OFF_ ≈ 10^8^) (Figs. S22 and S23). The values of the *V*_TH_ (Fig. 4D) were obtained via the second derivative method (Fig. S24), which showed a positive *V*_TH_ (*V*_TH_ = 1.71 ± 0.32 V). Notably, the E-mode FETs, which are completely turned off at *V*_GS_ = 0 V, were more favorable for constructing low-power consumption ICs than the D-mode transistors. The field-effect mobility of the transistors is a very important parameter for evaluating the current driving capability of the semiconductors, which can be calculated using the following formula: *μ*_FE_ = *g*_m_ × *L* / (*W* × *C*_g_ × *V*_DS_), where *L* and *W* are 10 and 50 μm, respectively. The gates’ capacitance was calculated using the following formula: *C*_g_ = *ε*_0_ × *ε*_r_ / *d*, where *ε*_0_ for Al_2_O_3_ is 9.0, *ε*_r_ is 8.85 × 10^−12^ F/m, and the thickness of Al_2_O_3_ is 30 nm. As shown in Fig. 4E, the average FET mobility was thus extracted as 34.28 ± 0.46 cm^2^ V^−1^ s^−1^ (the *g*_m_ results are shown in Fig. S25). Figure 4F shows a statistical analysis of the SS of the MoS_2_ FETs, which presented a relatively low SS (155.8 ± 33.7 mV dec^−1^). We also performed a detailed analysis of the uniformity in the electrical characteristics across the measured devices, as illustrated in Fig. S26. All the above results demonstrated that our MoS_2_ films were promising materials for future device applications.

**Fig. 5.**
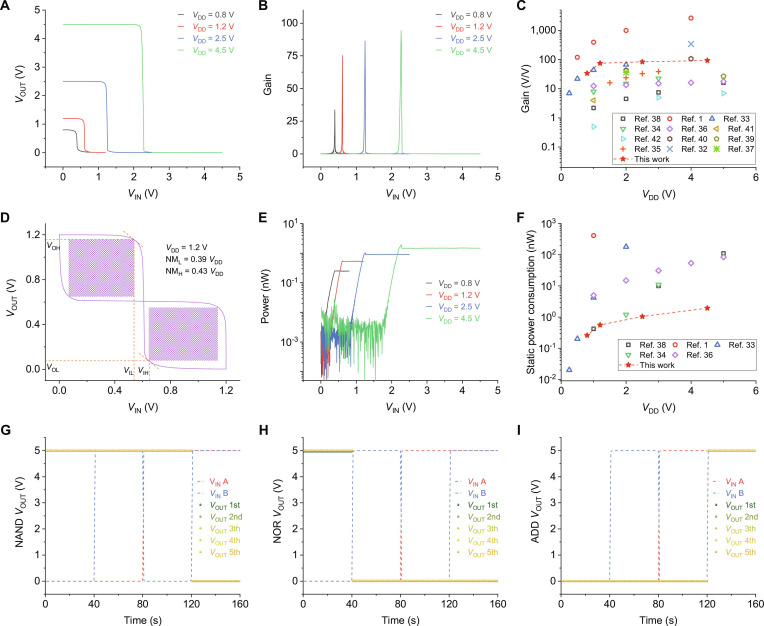
Inverter gate and logic circuits based on MoS_2_ single-crystal film. (A and B) Voltage transfer characteristics (A) and gain (B) of the MoS_2_ inverter. (C) Comparison of the voltage gain of our MoS_2_ inverter with other previously reported inverters based on MoS_2_. (D) Voltage transfer characteristics and the mirror reflection of the MoS_2_ inverter at *V*_DD_ = 1.2 V. (E) Power consumption of the inverter gate at different *V*_DD_. (F) Comparison of the power consumption of our MoS_2_ inverter with other previously reported inverters based on MoS_2_ (*V*_IN_ = 0 V). (G to I) Output characteristics of the NAND (G), NOR (H), and AND (I) gates.

Thanks to the successful fabrication of the E-mode FETs, some ICs, including the inverter, NAND, NOR, and AND circuits, were constructed. Figure S27 shows the optical images and schematics of these circuits, respectively. A series of voltage–transfer curves of the MoS_2_ demonstrated rail-to-rail outputs, highly sharp voltage transition (Fig. 5A), and high gain (Fig. 5B) at the supply voltage (*V*_DD_) from 0.8 to 4.5 V, where the gain reached up to 94 at a *V*_DD_ of 4.5 V, which is comparable to the previous reported TMD-based inverter gates (Fig. 5C) [1,32–42]. Noise margin is a key index to evaluate the performance of metal-oxide-semiconductor inverters, which is defined as (NM_L_ + NM_H_)/*V*_DD_, where NM_L_ (NM_L_ = *V*_IL_ − *V*_OL_) and NM_H_ (NM_H_ = *V*_OH_ − *V*_IH_) are the low- and high-level noise margins, respectively. Figure 5D gives the voltage–transfer curve and its mirror reflection of the MoS_2_ inverter at a *V*_DD_ of 1.2 V, which was used for extracting the NM_L_ and NM_H_. The calculated value of the noise margin reached up to 82%, which confirmed that the MoS_2_ inverters had high noise tolerance. The power consumption of these devices, defined as *P* = *V*_DD_ × *I*_DD_, where *P* is power consumption, and *I*_DD_ is supply current, is shown in Fig. 5E. The maximum power consumption was less than 0.3 nW at a *V*_DD_ of 0.8 V, which was attributed to the high-quality n-type MoS_2_ channel materials. As shown in Fig. 5F, our results exhibited lower power consumption compared with previously reported papers [1,33,34,36,38]. Table S2 gives the detailed performance comparison of devices in the references and this work. The other basic logic circuits based on the high-performance MoS_2_ inverters were fabricated. Figure 5G to I shows the output performance of the logic NAND (Fig. 5G), NOR (Fig. 5H), and AND (Fig. 5I) circuits. In our tests, *V*_IN_ A and *V*_IN_ B were the input signals, and we inputted signals (0, 0), (0, 1), (1, 0), and (1, 1), each lasting 40 s; “0” and “1” are 0 and 5 V, respectively. All the logic circuits were tested 5 times and showed rail-to-rail outputs with excellent stability. Table S3 gives the corresponding truth table. The output waveforms of these circuits are presented in Fig. S28, demonstrating well-behaved operations.

## Discussion

In summary, the centimeter-scale MoS_2_ single-crystal films were successfully synthesized, using a well-designed sandwiched Mo source, reducing the growth time to just 10 min. The sulfur vacancy defect density in the MoS_2_ film was measured to be 8.49 × 10^12^ cm^−2^. The designed MoS_2_ FETs exhibited high on-state current (6.54 ± 0.82 μA/μm at *V*_DS_ = 1 V), low off-state current, high on–off ratio (>10^8^), positive *V*_TH_ (1.71 ± 0.32 V), high mobility (34.28 ± 0.46 cm^2^ V^−1^ s^−1^), and low SS (155.8 ± 33.7 mV dec^−1^). Inverter gates based on the high-performance E-mode MoS_2_ FETs showed high gain, high noise margins, and low power consumption. Furthermore, logic circuits including NAND, NOR, and AND gates were fabricated and exhibited rail-to-rail outputs. Our results not only provide an effective approach for growing MoS_2_ films with ultrashort growth times but also demonstrate a great application for future ICs.

## Materials and Methods

### Substrate and Mo source preparation

Before synthesis, a C/A miscut sapphire (diameter: 4 inches) was annealed at 1,040 °C for 4 h. The annealed sapphire was cut into 2-cm squares and cleaned in isopropyl alcohol, acetone, and deionized water to remove surface impurities, respectively. MoO_3_ (0.5 g, 99.99%) and NaCl (0.05 g, 99.9%) powders were mixed and evenly placed on the top surface of a Mo foil (99.95%, 0.025 mm thick, 2 cm × 2 cm). Another Mo foil with the same specification was placed on top of the above-mentioned Mo foil. After that, the Mo foil/MoO_3_ + NaCl/Mo foil sandwich metal source was prepared.

### Growth of MoS_2_

The MoS_2_ samples were synthesized by using a one-zone tube furnace (HF-Kejing, OTF-1200F-X, diameter: 2 inches). A ceramic crucible containing sublimed sulfur powder (10 g, 99%) loaded into a 2-inch CVD chamber was placed 14 cm upstream from the center. As shown in Fig. 1A, a rectangular graphite boat (2 cm × 6 cm) with the sapphire substrate and the sandwiched Mo source was placed in the chamber’s center. The sapphire substrate was placed at the head position of the graphite boat, and the distance between the sapphire substrate and the Mo source was controlled at 2 cm. High-purity argon (Ar) from upstream to downstream was used as carrier gas. The gas flow was first set as 1,200 standard cubic centimeters per minute (SCCM) for 10 min before heating for cleaning the chamber, and then set as 75 SCCM for the growth of the MoS_2_ at atmospheric pressure. The furnace was heated to 930 °C within 30 min. The growth was performed for less than 2 min to obtain single-oriented MoS_2_ monolayers, and for 10 min to obtain the centimeter-scale (2 cm × 2 cm) MoS_2_ films. Finally, the furnace was cooled to room temperature automatically.

### Transfer of MoS_2_ films

Polymethyl methacrylate (PMMA, 950K, 4A) was spin-coated on the MoS_2_/sapphire substrate at 4,000 rpm and baked at 180 °C for 90 s. A TRT (2 cm × 2 cm) was pasted on PMMA/MoS_2_/sapphire and soaked in 10% KOH for 5 min. The TRT/PMMA/MoS_2_ was peeled off from the sapphire and transferred onto the target substrate, and baked at 120 °C to release the TRT. PMMA was removed by acetone (Fig. S8). The MoS_2_ monolayer was prepared for TEM analysis using a 2-step transfer process. The first step involved transferring the MoS_2_ monolayer from the original sapphire substrate onto a SiO_2_/Si (100) substrate and only releasing the TRT film, as described above. Then, the SiO_2_/Si substrate with the PMMA/MoS_2_ film was cut into small square pieces measuring about 3 mm. The sample was immersed in a 10% KOH solution for 2 h to wet-etch the SiO_2_ layer, allowing the PMMA/MoS_2_ film to detach and float on the surface. The PMMA/MoS_2_ film was washed 3 times with deionized water and then scooped up with a copper grid. After drying, PMMA is removed with acetone (Fig. S9).

### Characterization

The morphologies of all samples were characterized by optical microscopy (Zeiss Axio Scope A1), AFM (Bruker Multimode 8), and STEM (FEI Themis Z 3.2 operated at 300 KV). PL and Raman measurements were performed by a confocal microscope (WITec, alpha-300) with a 532-nm diode continuous-wave laser as the excitation. Absorption spectra were obtained by a modified Olympus microscope system, and a tungsten lamp (Zeiss HAL 100) was used as the light source. The light source was focused by an objective lens (50×, Zeiss, 0.75NA). The absorbance (*A*) was calculated as *A* = 1 – *I*_0_/*I*, where *I* and *I*_0_ are the light intensities transmitted through the quartz substrate on and off the samples, respectively. The SHG experiments were performed in the Olympus microscope system. A 1,030-nm pulse laser (80 MHz repetition rate) was focused by the objective lens (50×, Zeiss, 0.75 NA), and the SHG signal was collected by the same objective. A650 short-pass dichroic mirror was applied to filter the excitation laser.

### Device fabrication and measurement

For the device fabrication, the back gate 10 nm Bi/20 nm Au metal array was fabricated on a bare 300-nm SiO_2_/Si wafer by using lithography and thermal evaporation. Then, a 30-nm Al_2_O_3_ dielectric layer was deposited by using ALD and patterned by using lithography and inductively coupled plasma reactive ion etching (ICP-RIE). After that, the MoS_2_ film on the Al_2_O_3_ substrate was transferred onto the target substrate using the wet transfer method. The MoS_2_ film on the new substrate was patterned by using lithography and ICP-RIE. Finally, the source and drain 10- to 15-nm Bi/50 nm Au metal array was fabricated by using lithography and thermal evaporation. The electrical properties of the FETs and logic circuits of the MoS_2_ films were measured in vacuum (∼10^−5^ Pa) with a Lake Shore Probe Station and an Agilent B1500A semiconductor analyzer at room temperature.

## Data Availability

All data are available in the manuscript or the Supplementary Materials, or from the authors.
