# Supplementary material for: Fast Growth of Centimeter-Scale Molybdenum Disulfide Single Crystal for Energy-Efficient Logic Circuits
Source: Research (Wash D C). 2026 Jan 28;9:1117. doi: 10.34133/research.1117 (PMC12848893; doi:10.34133/research.1117)
Supplement: Supplementary 1 — Figs. S1 to S28 Tables S1 to S3 [file research.1117.f1.docx]

Supplementary Materials for

**Fast growth of centimeter-scale molybdenum disulfide single crystal for energy-efficient logic circuits**

Biyuan Zheng^1,2†^, Hui Wang^1†^, Yizhe Wang^1†^, Weihao Zheng^2†^, Yong Liu^1^, Guangcheng Wu^1^, Miaomiao Li^2^, Sha Wang^1^, Xingxia Sun^1^, Chenguang Zhu^1^, Xin Yang^1^, Zheyuan Xu^1^, Mengjian Zhu^2^, Li Xiang^1*^, Dong Li^1*^, Anlian Pan^1,3*^

^1^Hunan Institute of Optoelectronic Integration, College of Materials Science and Engineering, Hunan University, Changsha 410082, China. ^2^College of Advanced Interdisciplinary Studies & Hunan Provincial Key Laboratory of Novel Nano-Optoelectronic Information Materials and Devices, National University of Defense Technology, Changsha, 410073, China. ^3^School of Physics and Electronics, Hunan Normal University, Changsha 410081, China

^*^Address correspondence to: xiangli93@hnu.edu.cn (L.X.); liidong@hnu.edu.cn (D.L.); anlian.pan@hnu.edu.cn (A.P.)

^†^These authors contributed equally to this work

**Table of Contents**

[Supplementary Figs. 2](#_Toc218606708)

[Supplementary Tables 16](#_Toc218606709)

# Supplementary Figs.


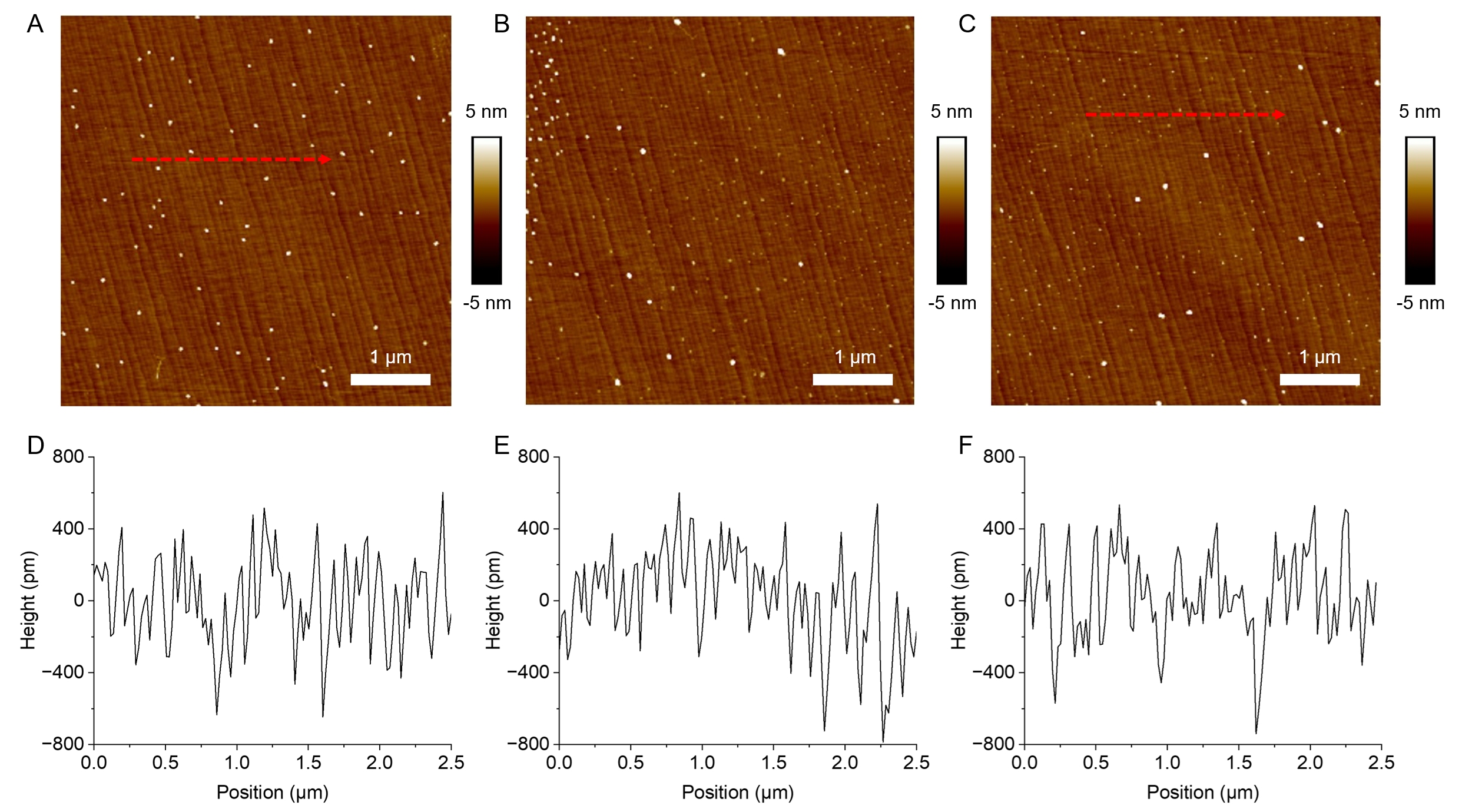


**Fig. S1.** (A to C) AFM images of the sapphire substrate collected from three positions. (D to F) Line scan height profiles along the red arrow in A to C.


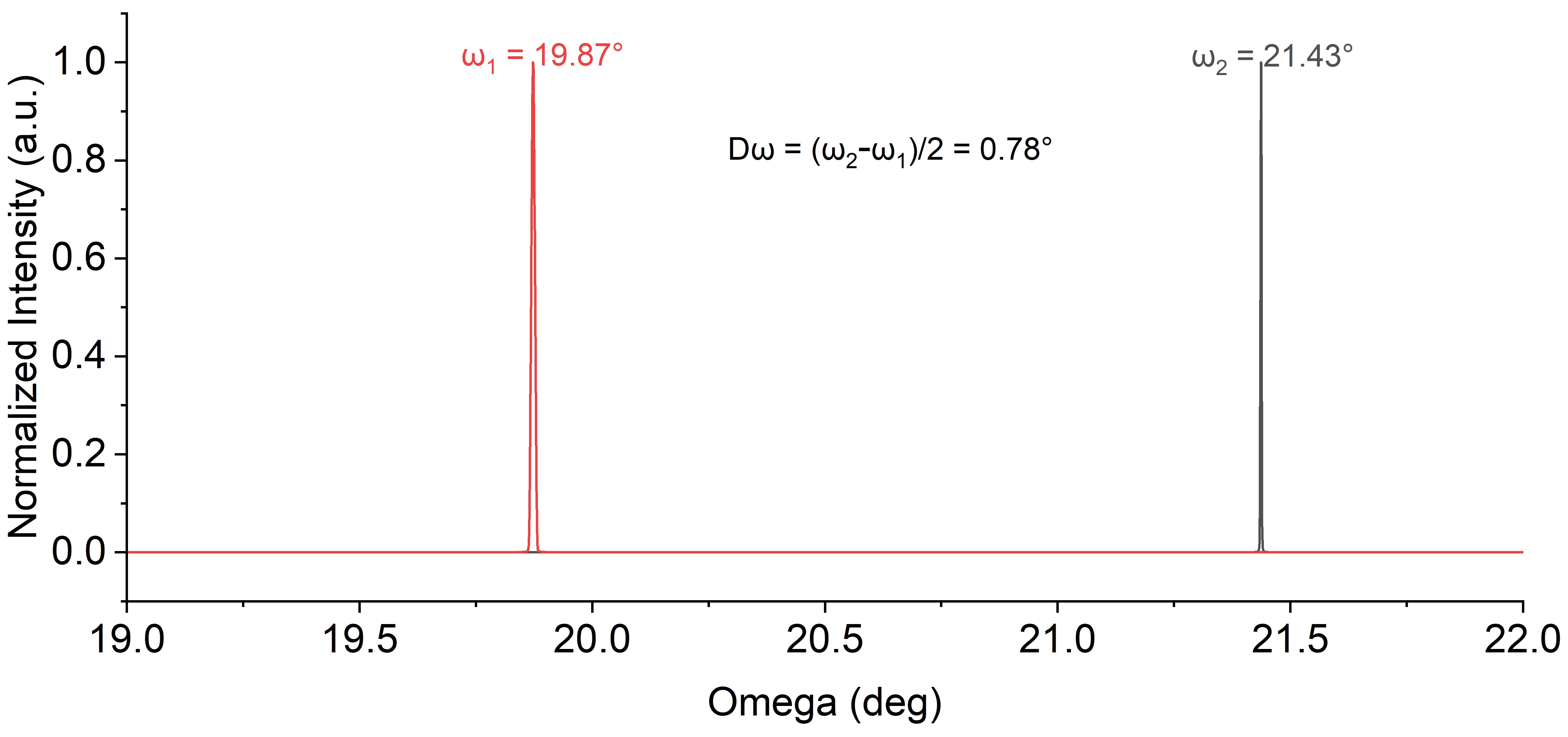


**Fig. S2.** XRD rocking curves of the sapphire substrate, demonstrating that the miscut angle is 0.78°.


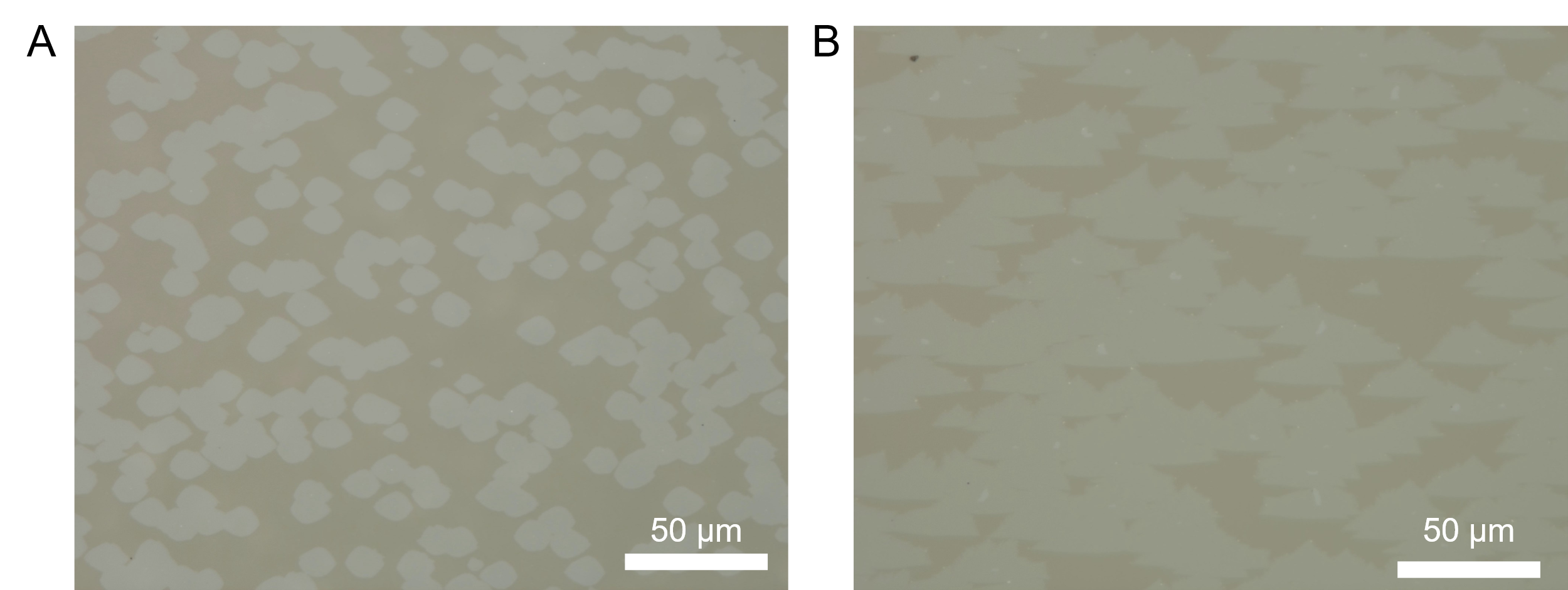


**Fig. S3.** (A and B) MoS_2_ domains with different morphologies.


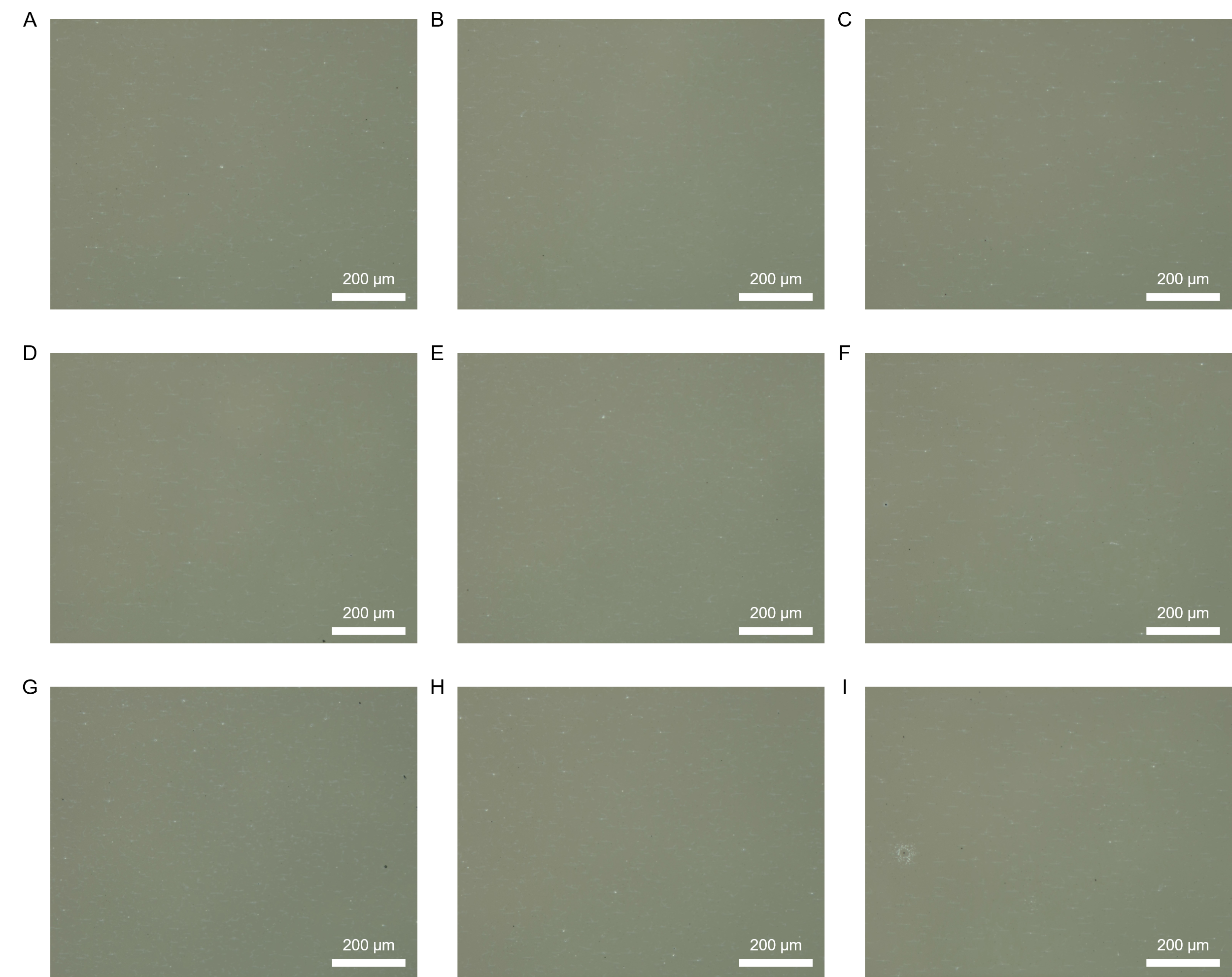


**Fig. S4.** (A to I) Optical images of MoS_2_ single crystal at random 9 positions.


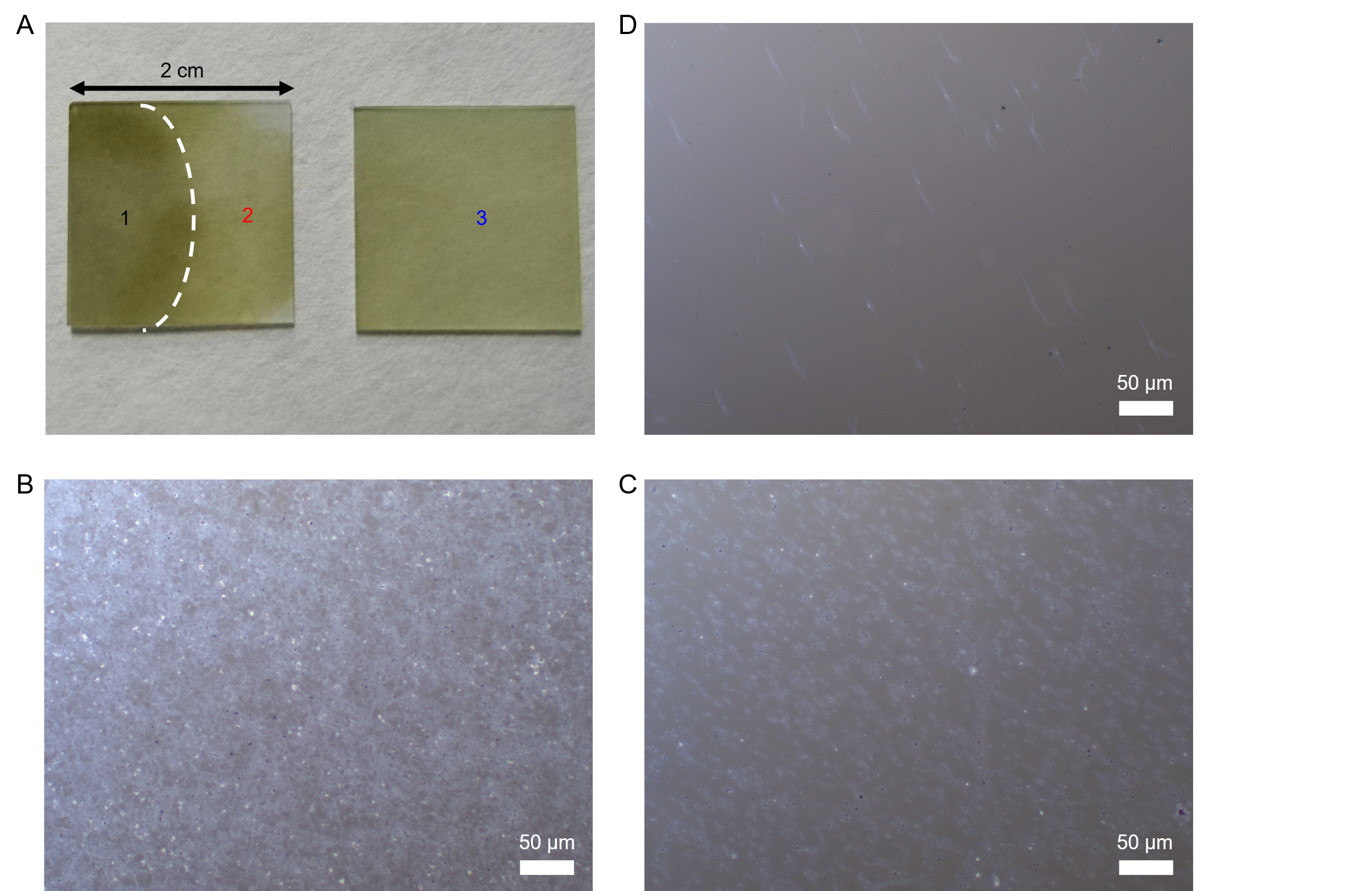


**Fig. S5.** (A) Photographs of MoS_2_ samples with the substrates at downstream (left sample) and upstream (right sample) positions of the Mo source. (B to D) Optical images of MoS_2_ films were obtained from point 1 (B), point 2 (C), and point 3 (D) in A.


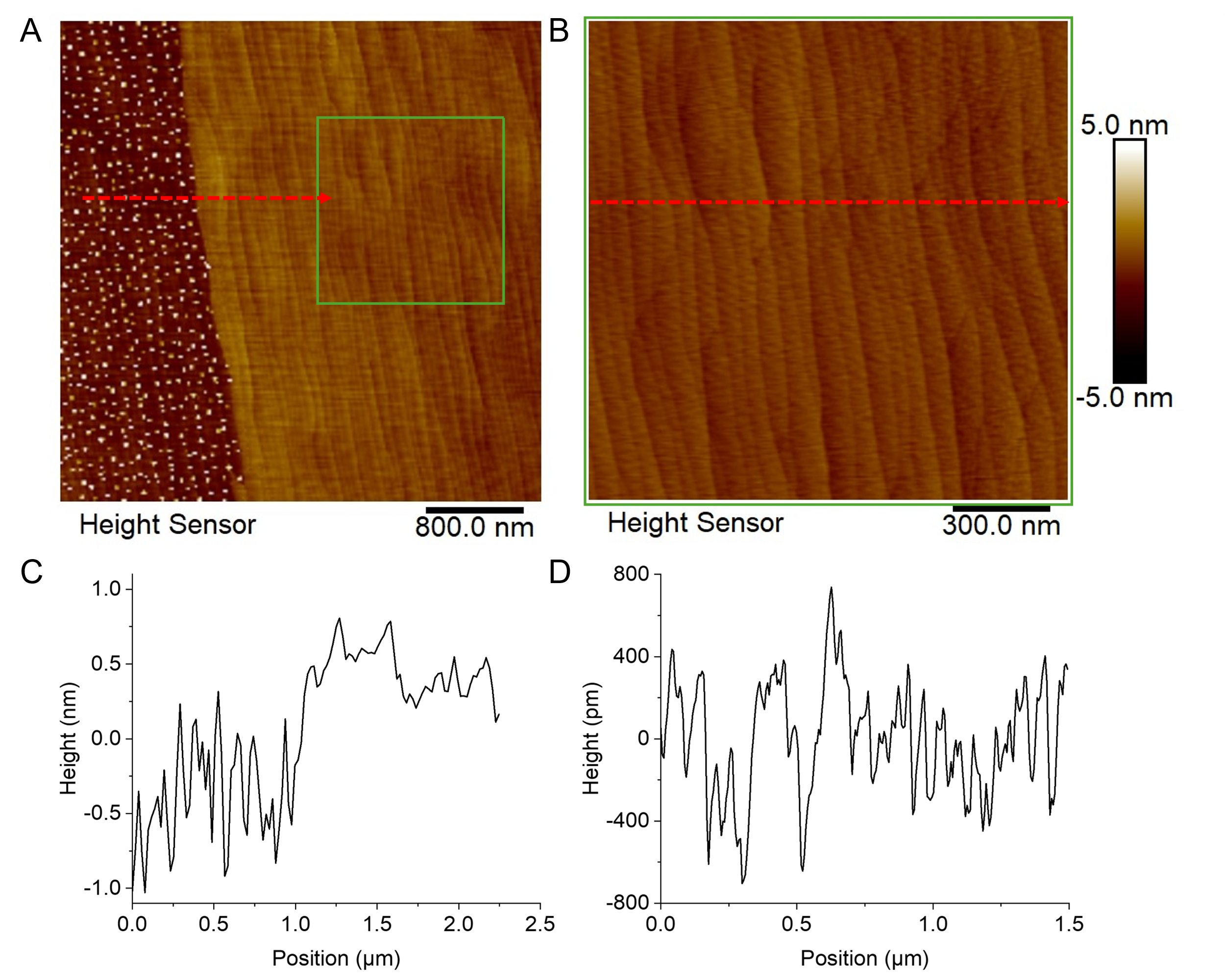


**Fig. S6.** (A) AFM image of the MoS_2_ grown on sapphire. (B) Magnified AFM image from the green rectangular area in (A). (C) Line scan height profile along the red arrow in (A). (D) Line scan height profile along the red arrow in (B).


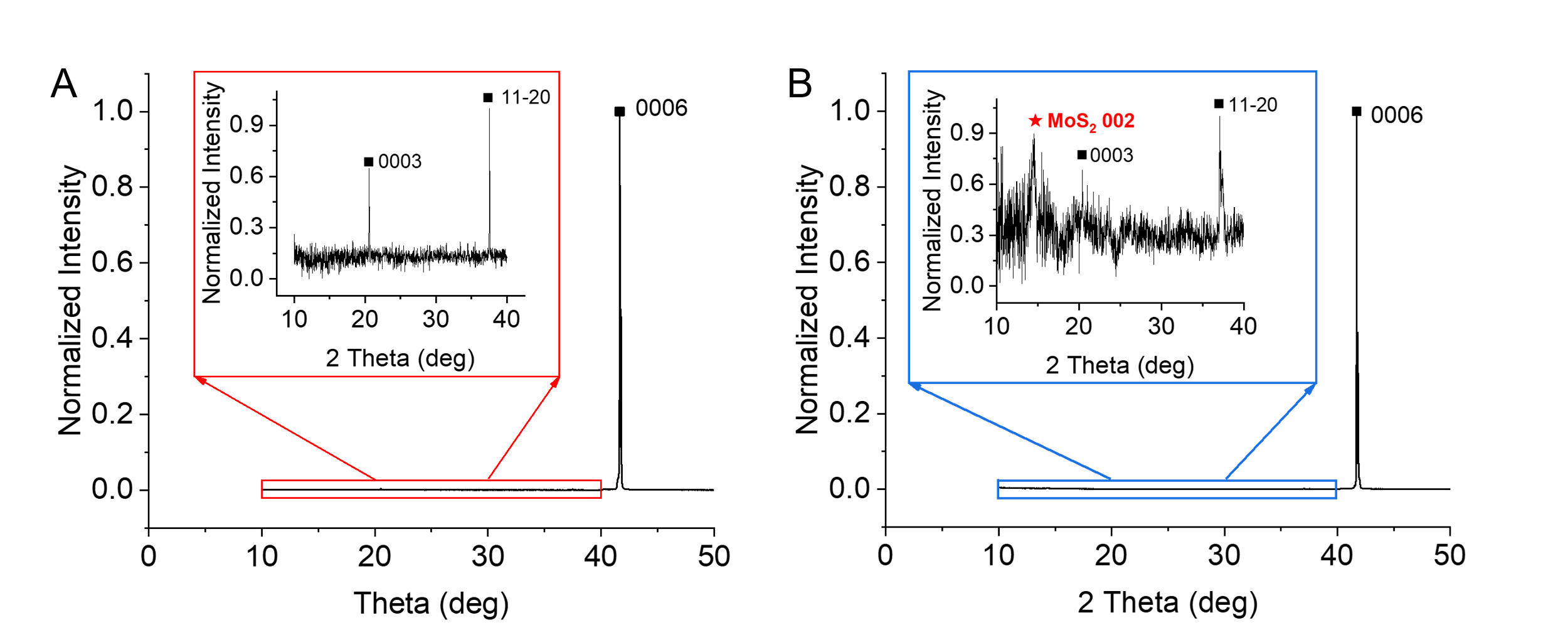


**Fig. S7.** (A and B) XRD pattern of the bare sapphire substrate (A) and MoS_2_ single crystal film on sapphire substrate.


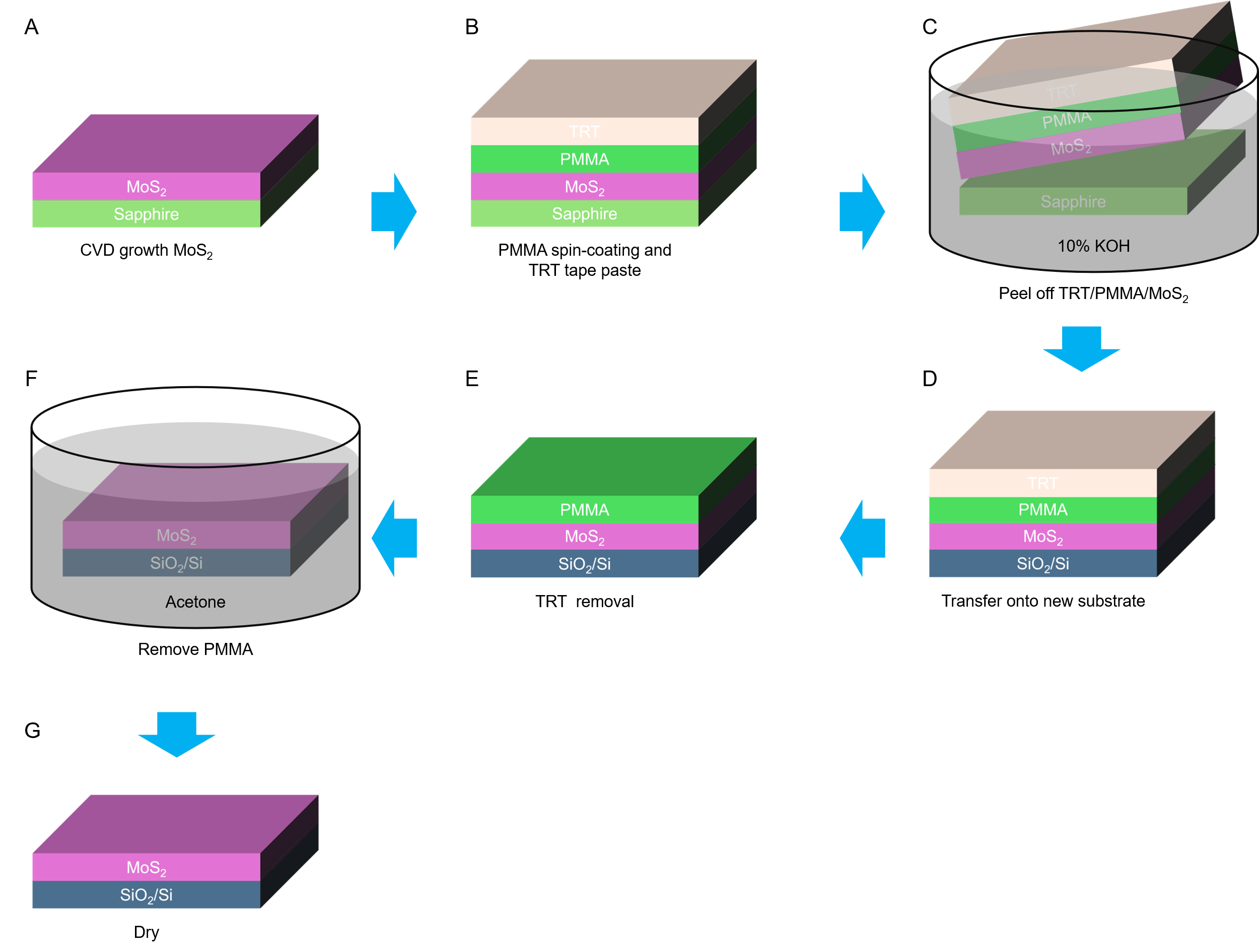


**Fig. S8.** (A to G) TRT-assisted wet transfer process of MoS_2_ films from sapphire to the target substrate (using SiO_2_/Si substrate as an example).


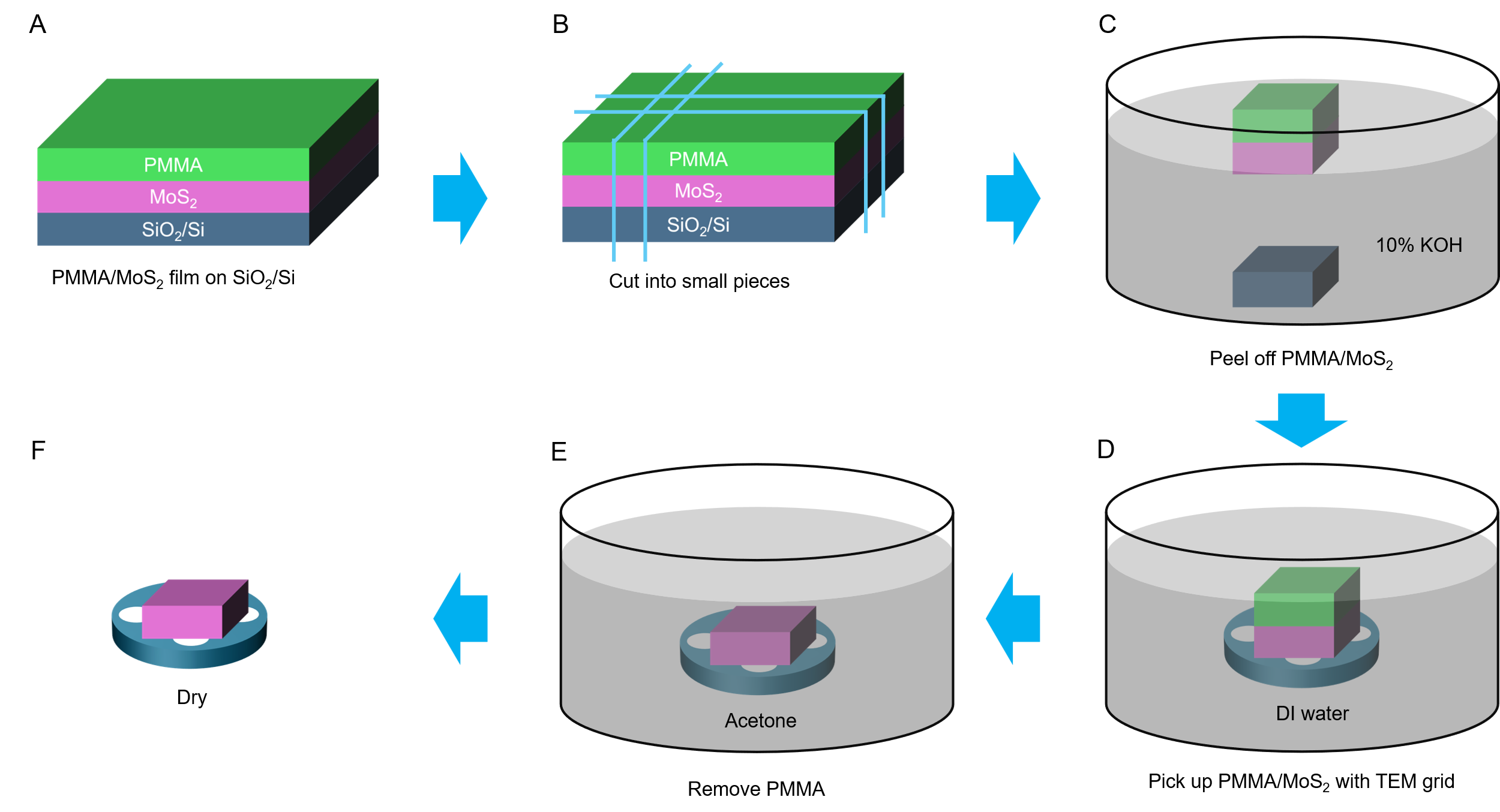


**Fig. S9.** (A to F) Wet transfer process of MoS_2_ films from SiO_2_/Si substrate to the TEM grid.


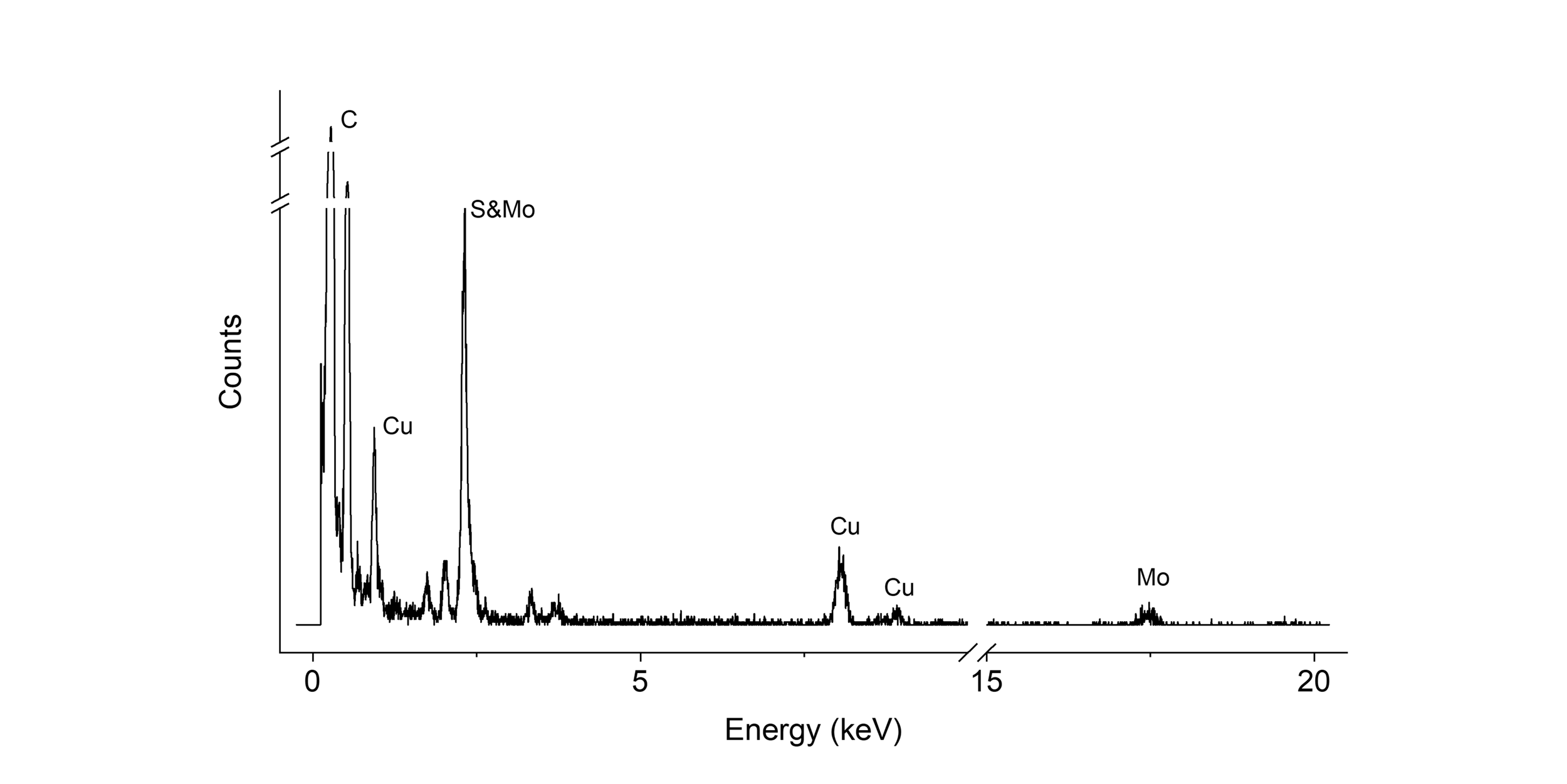


**Fig. S10.** STEM-EDS single spectra of the MoS_2_ monolayer.


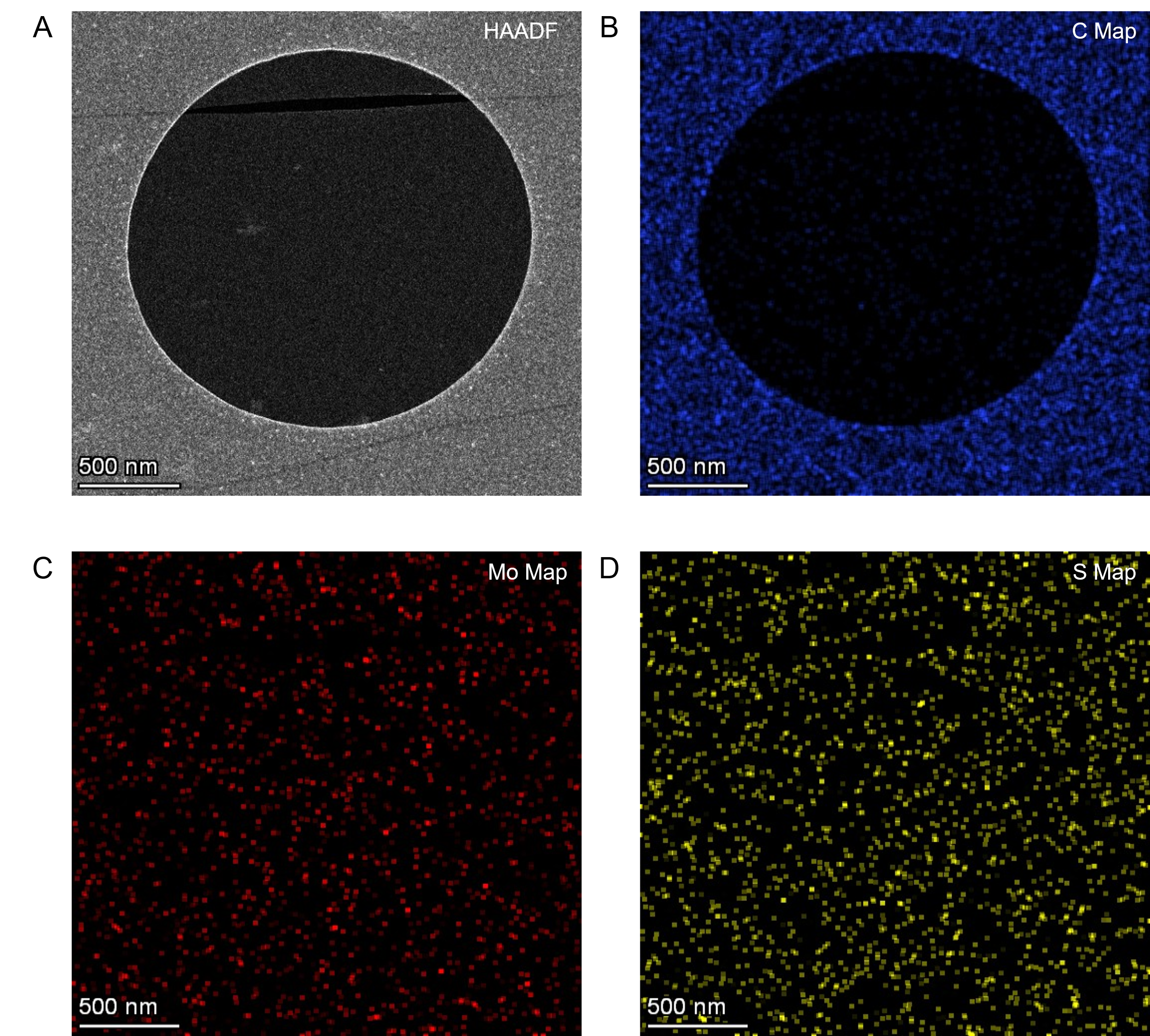


**Fig. S11.** (A to D) HAADF image and the corresponding C (B), Mo (C), and S (D) elements distribution profiles of MoS_2_ on the quantifoil holey carbon support.


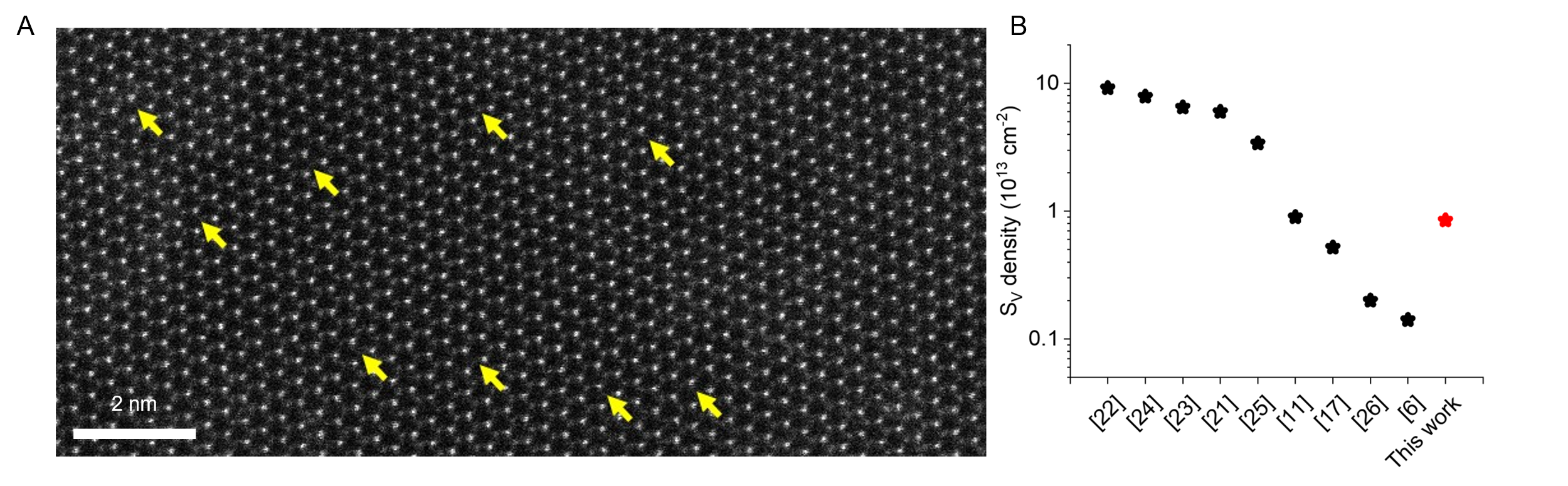


**Fig. S12.** (A) The HRSTEM image of MoS_2_ indicates high quality. (B) Comparison of sulfur vacancy density of our MoS_2_ film with other previously reported MoS_2_.


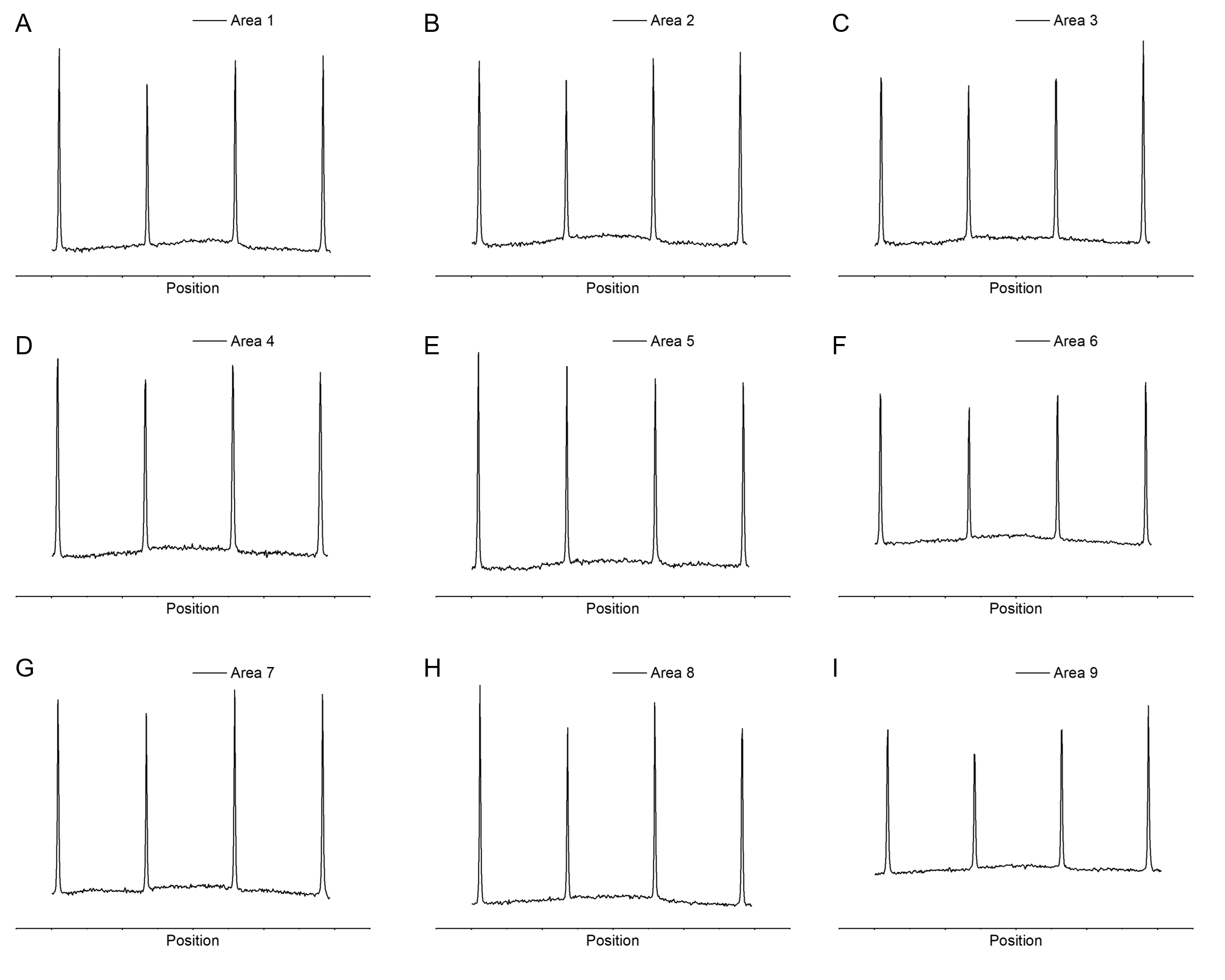


**Fig. S13.** (A to I) Line scan profiles through experimentally measured diffraction spots in Fig. 2G.


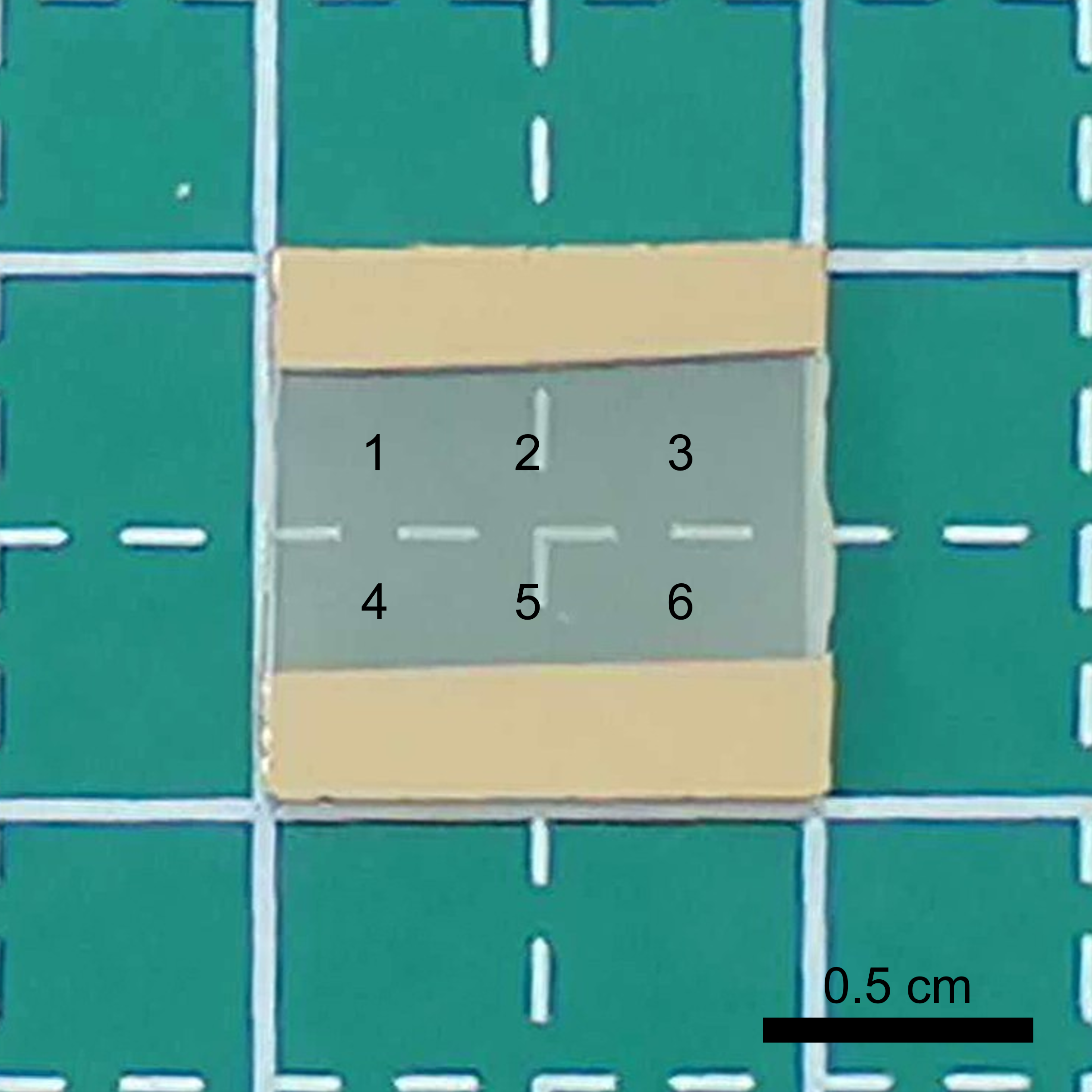


**Fig. S14.** Photograph of the centimeter-scale MoS_2_ single crystal film for the LEED test.


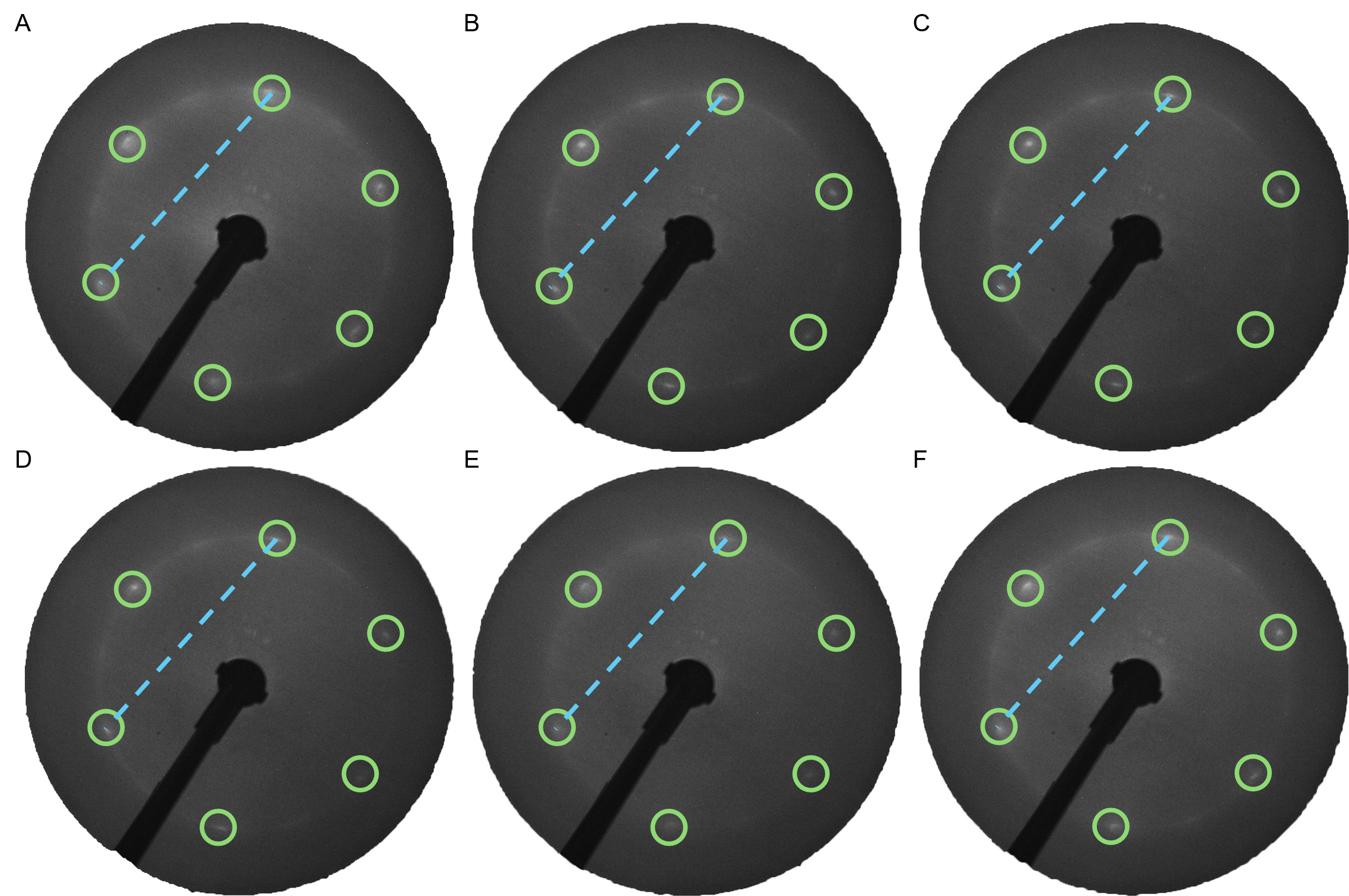


**Fig. S15.** (A to F) LEED patterns of the MoS_2_ single crystal film selected from points 1-6 in Fig. S14, taken at 90 eV. Circles, diffraction spots; dashed auxiliary lines, showing that the obtained MoS_2_ film was a single crystal.


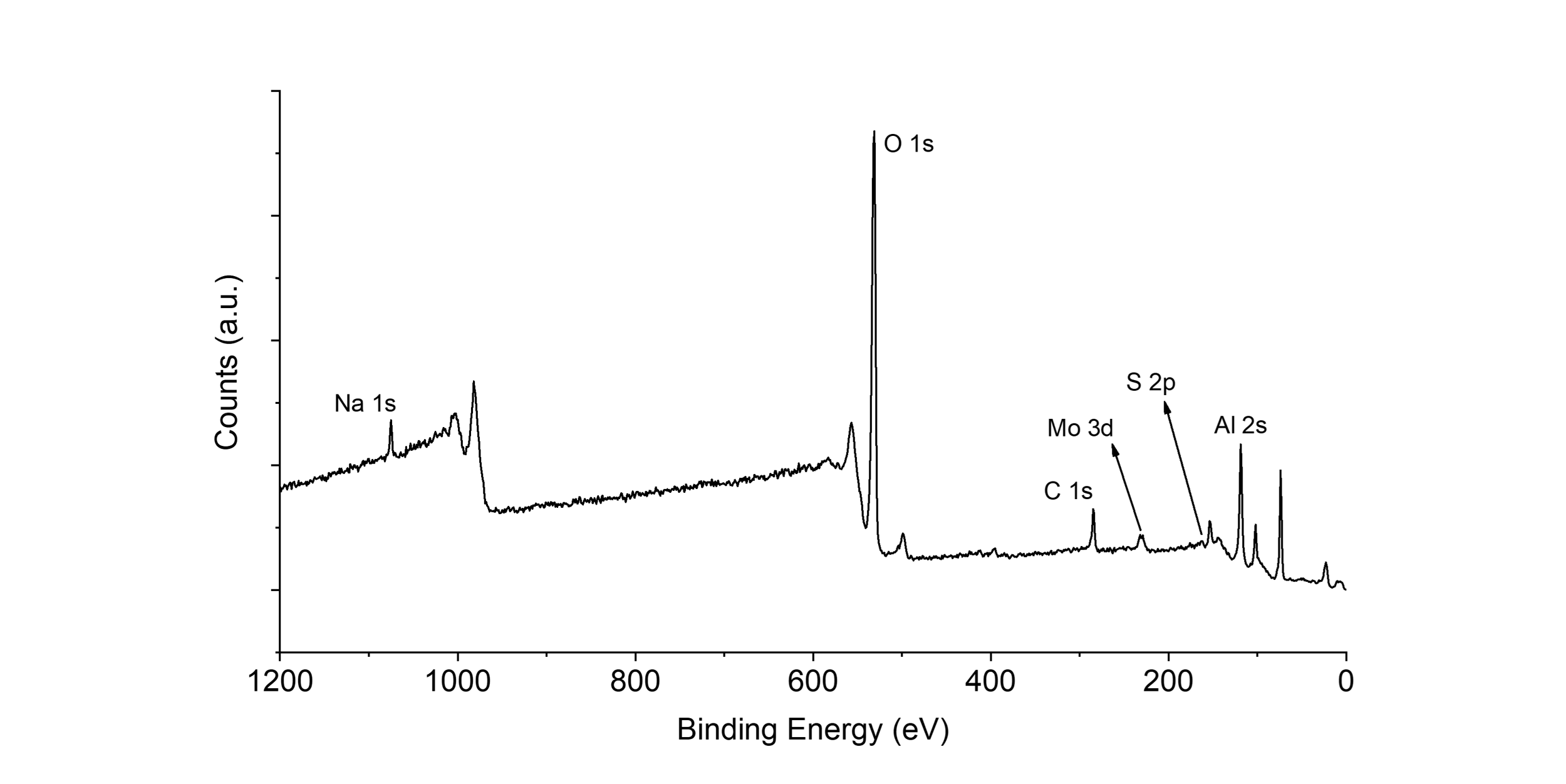


**Fig. S16.** XPS full spectra of single crystal MoS_2_ film on Al_2_O_3_ substrate.


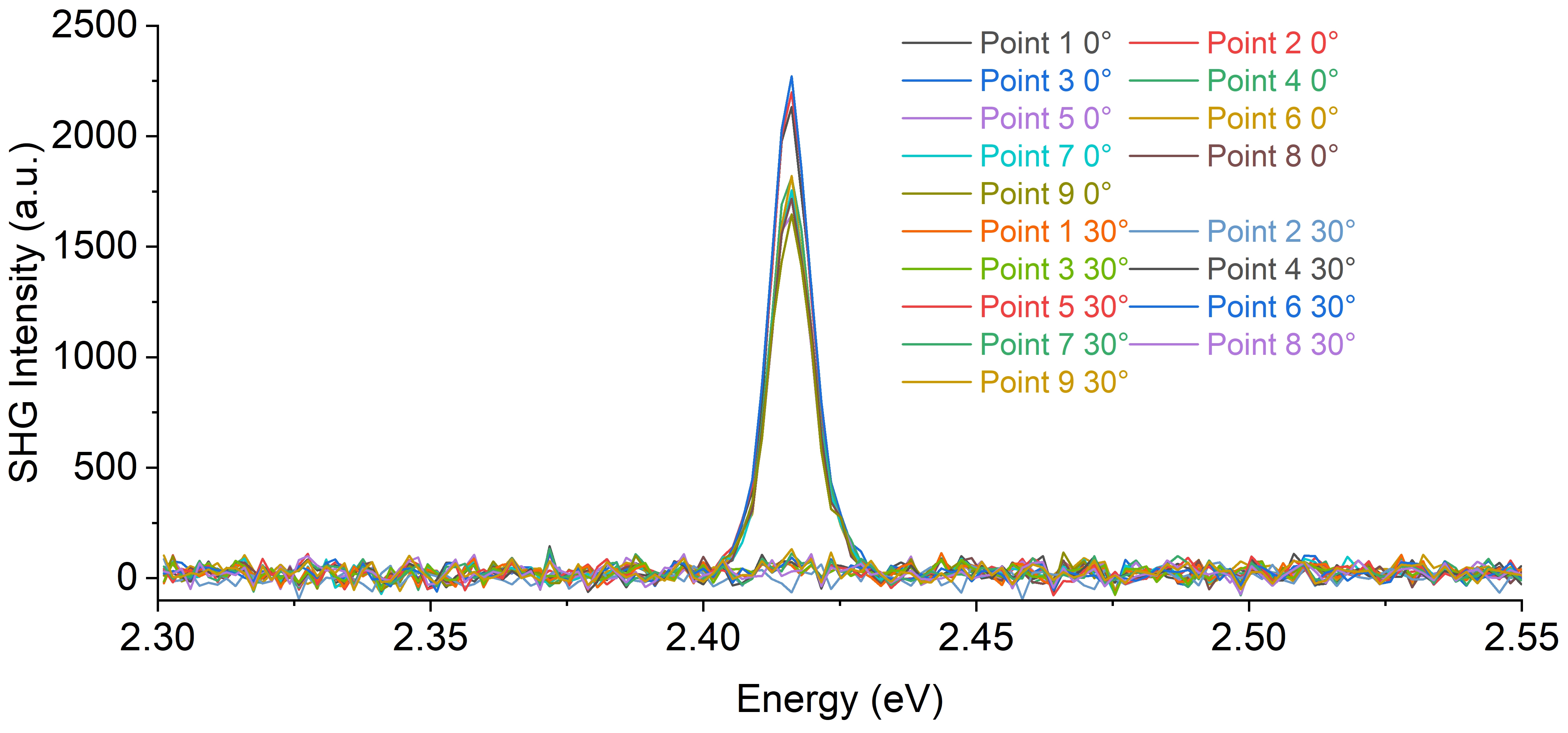


**Fig. S17.** SHG intensities of the detected samples with parallel-polarized laser angles at 0° and 30°.


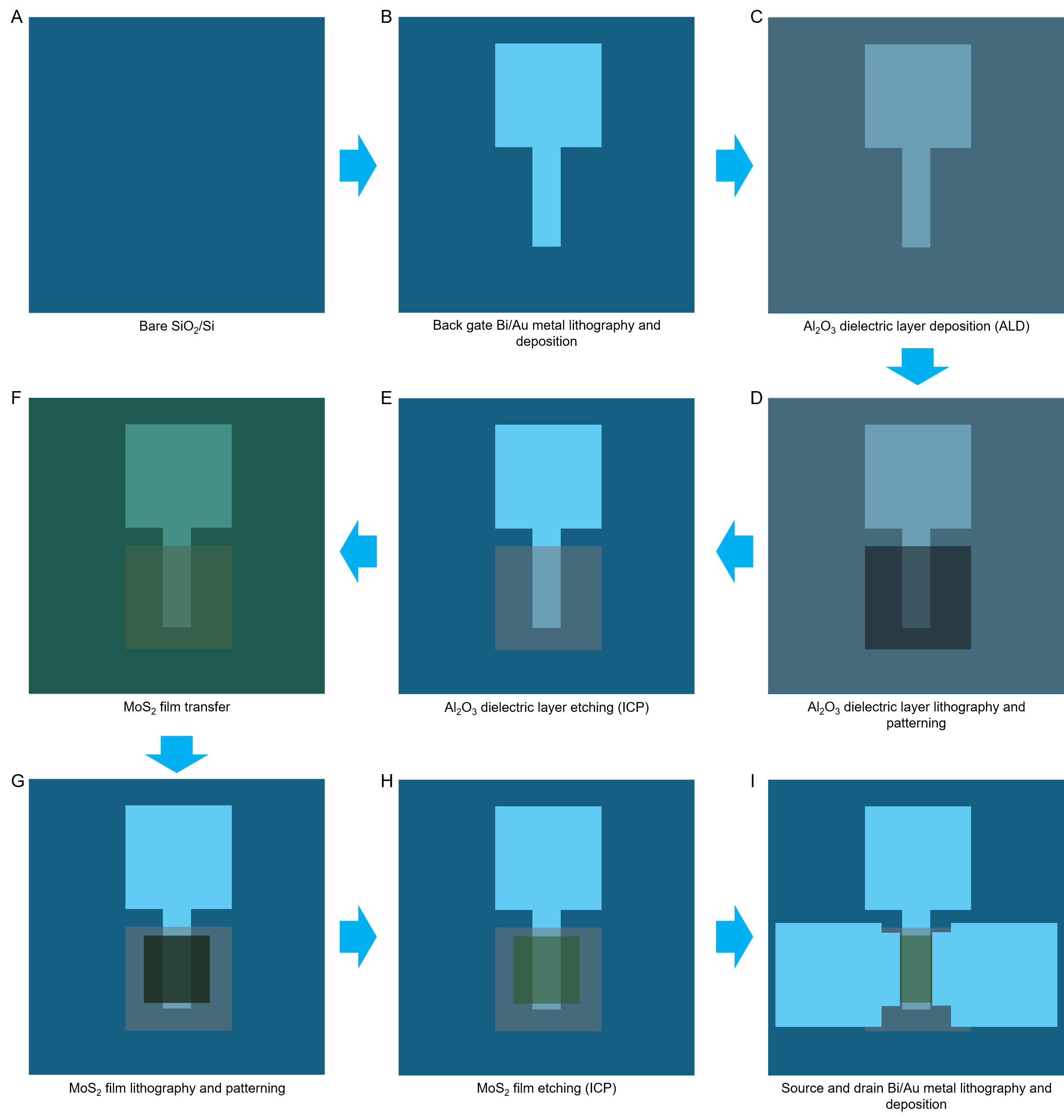


**Fig. S18.** (A to I) Fabrication process flow of FETs.


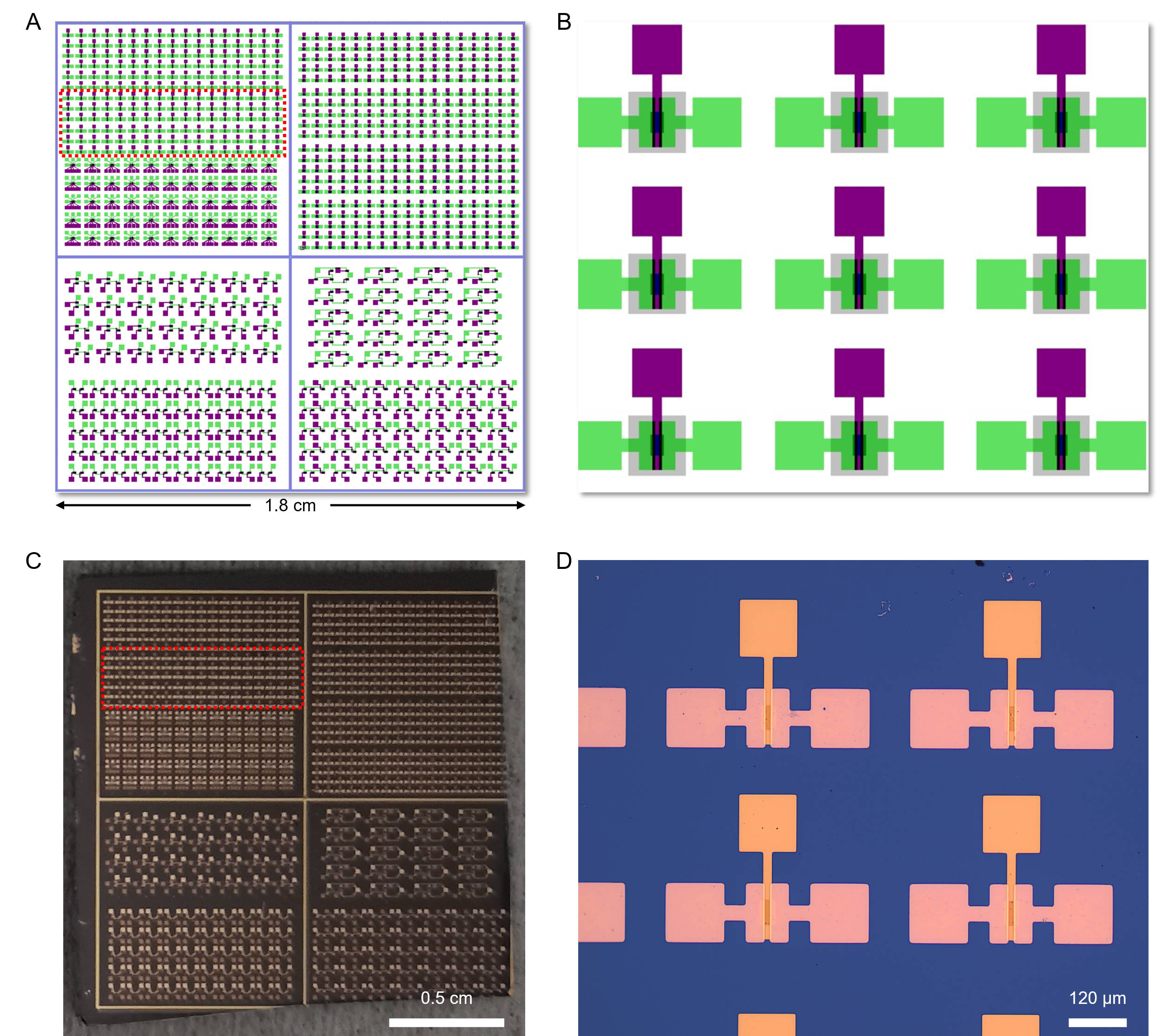


**Fig. S19.** (A) Device layout overview. (B) Detailed layout of a representative MoS_2_ FETs. (C) Photograph of the fabricated devices based on a continuous centimeter-scale MoS_2_ single crystal film. (D) Optical image of MoS_2_ FETs. The regions containing the 100 tested FETs are highlighted by red dashed boxes in (A and C).


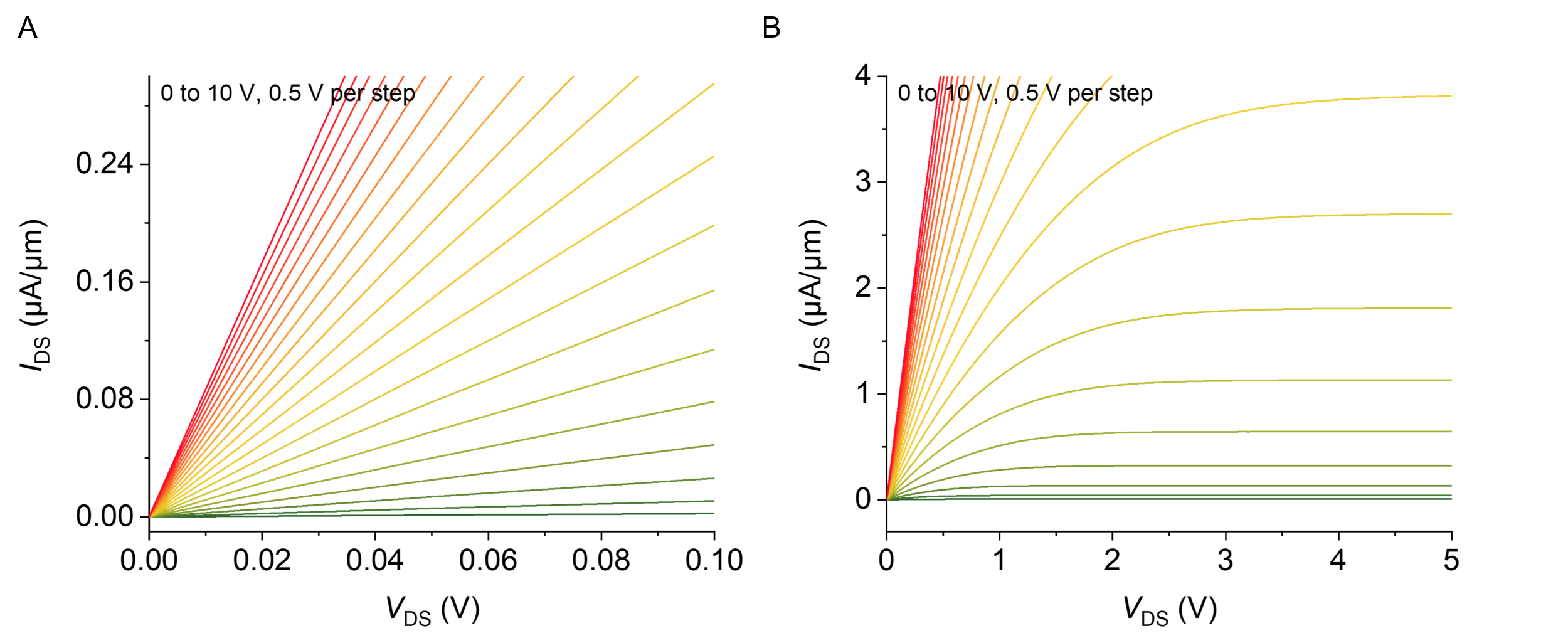


**Fig. S20.** (A and B) Output curves of the MoS_2_ FET at low (A) and high (B) bias, respectively.


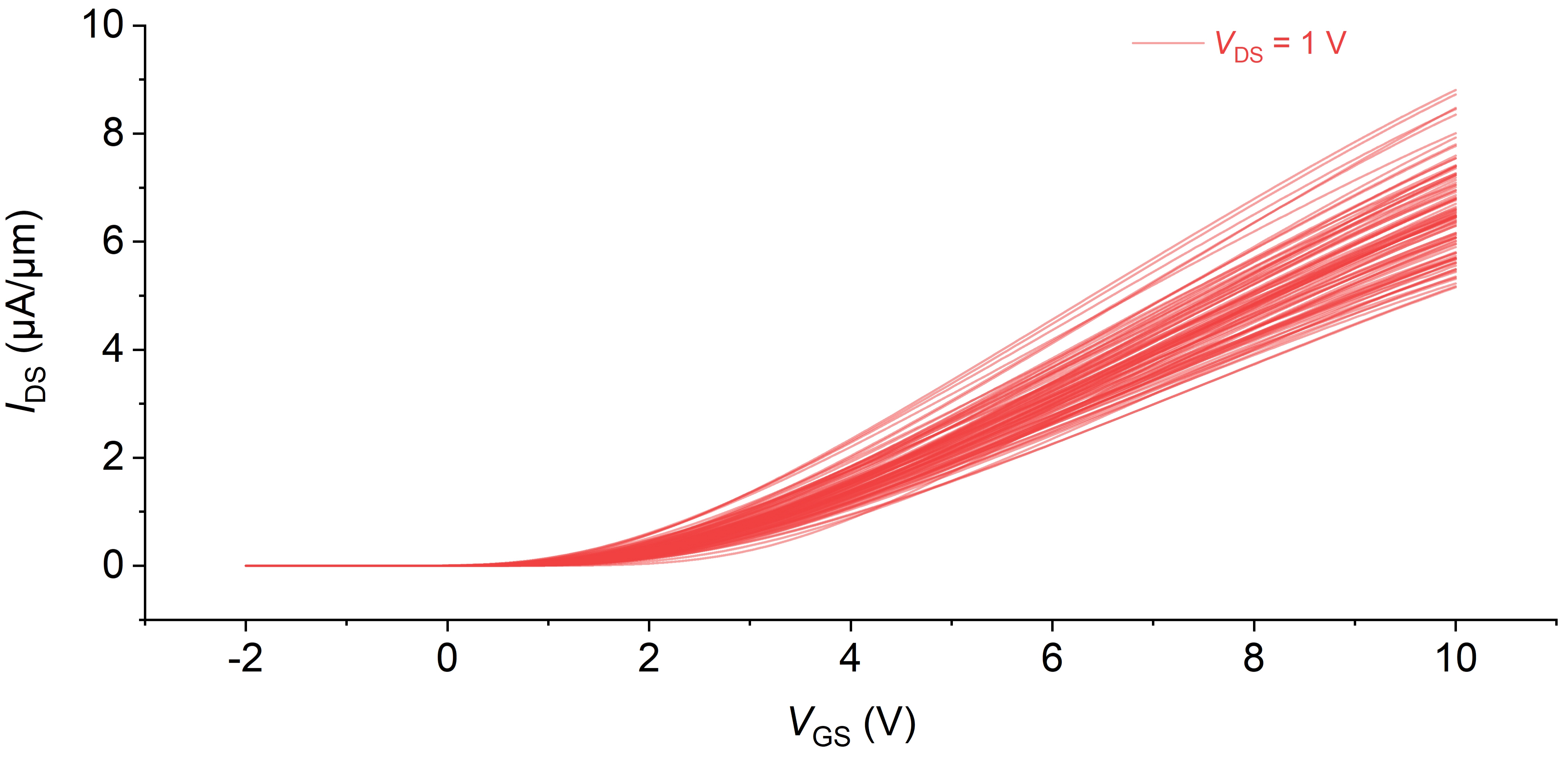


**Fig. S21.** Transfer curves of 100 MoS_2_ FETs at *V*_DS_ = 1V with linear Y scale.


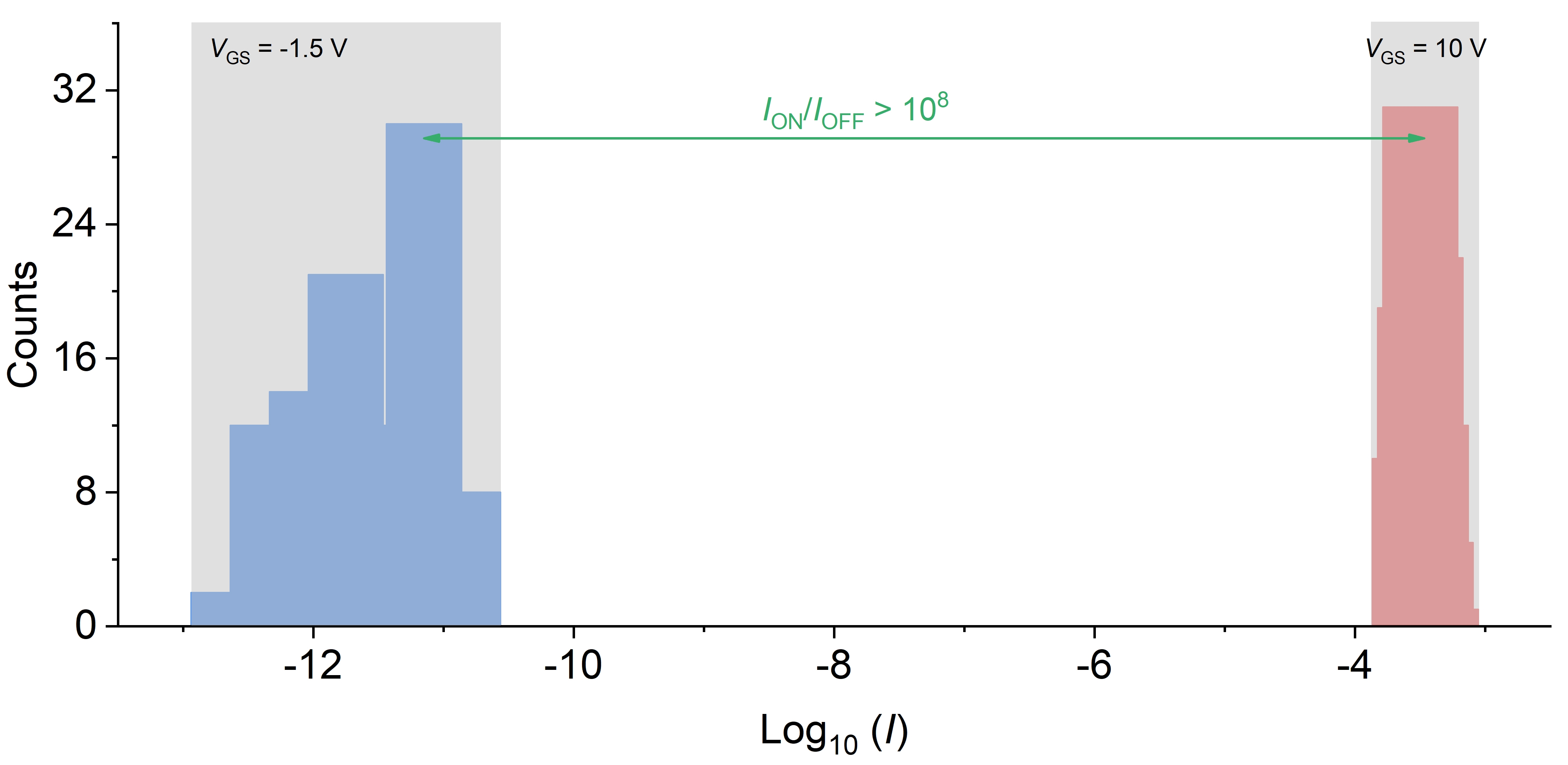


**Fig. S22.** Statistical distribution of on-state and off-state current of the 100 MoS_2_ FETs.


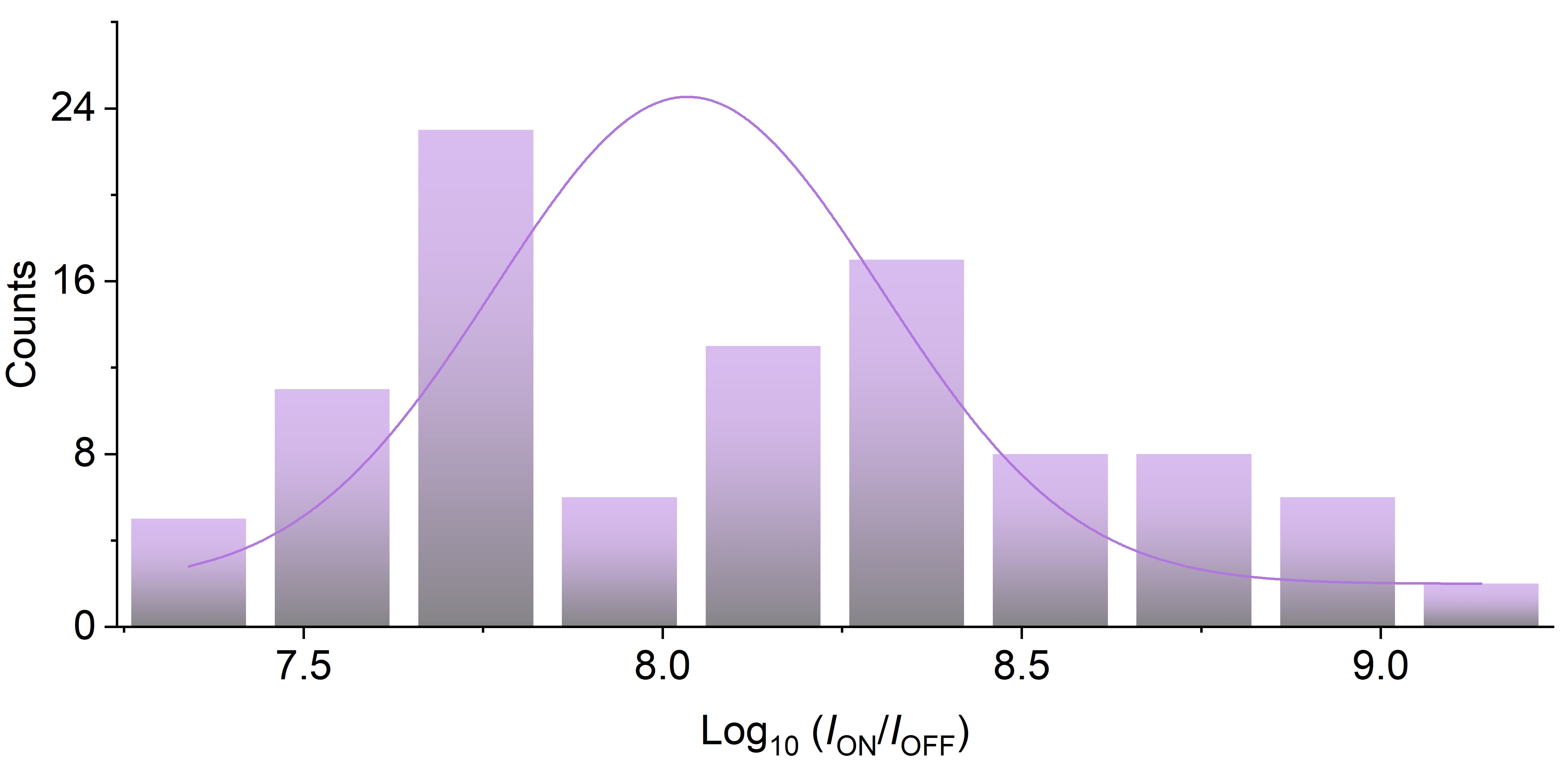


**Fig. S23.** Statistical distribution of the on-off ratio of the 100 MoS_2_ FETs.


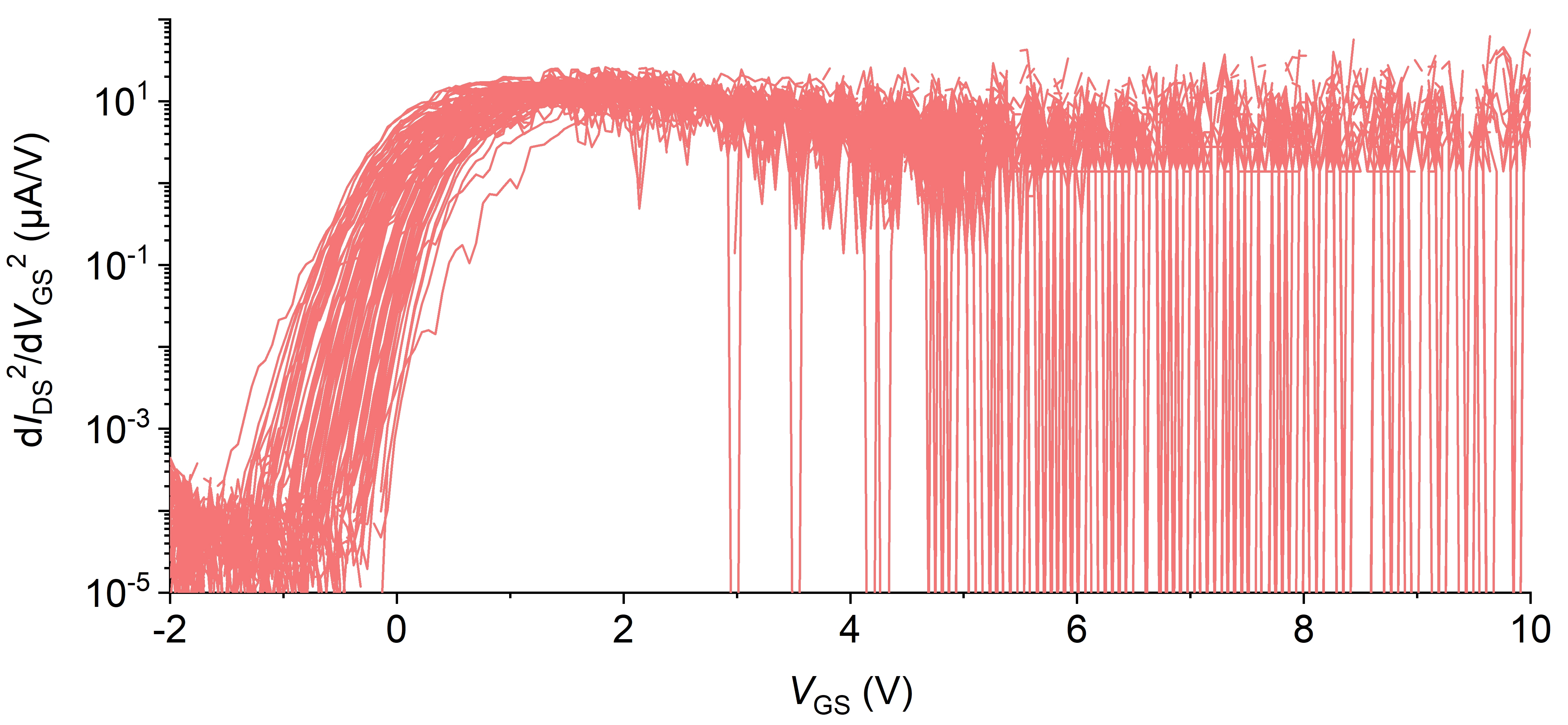


**Fig. S24.** The plots of d*I*_DS_^2^/d*V*_GS_^2^ versus *V*_GS_ of the 100 FETs were used to calculate the *V*_TH_.


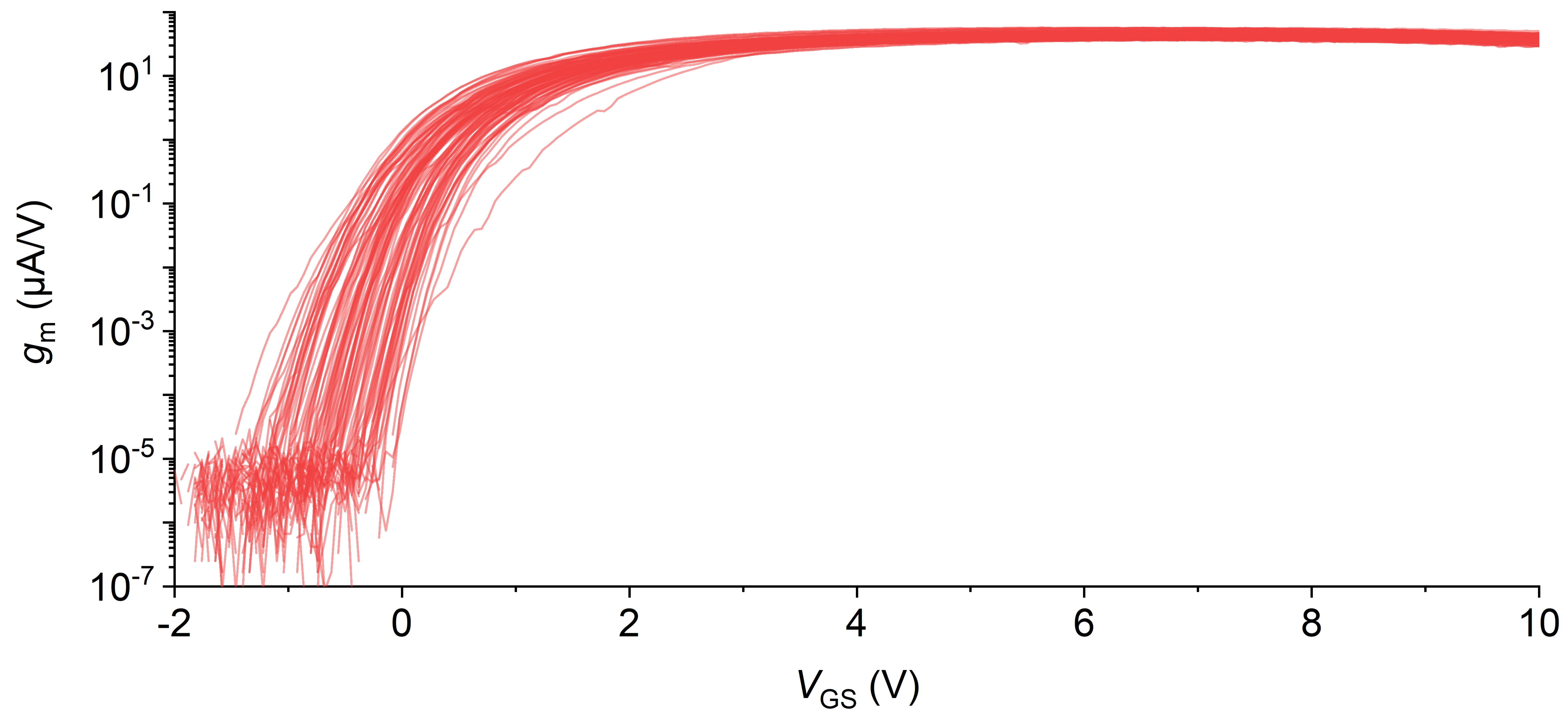


**Fig. S25.** The plots of *g*_m_ versus *V*_GS_ of the 100 FETs were used to calculate the mobility.


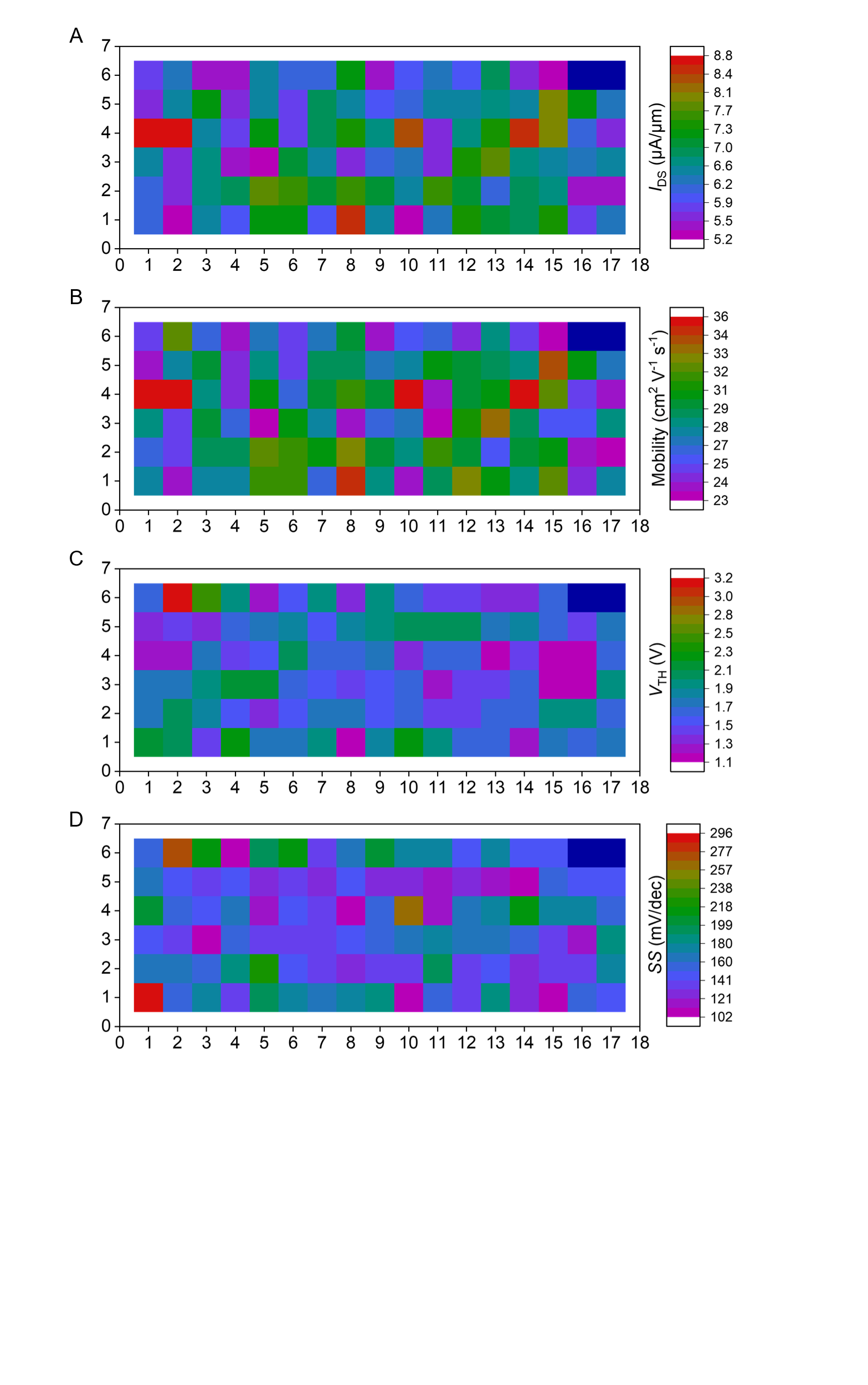


**Fig. S26.** (A to D) *I*_DS_ (A), mobility (B), *V*_TH_ (C), and *SS* (D) mapping of the 100 MoS_2_ FETs.

**
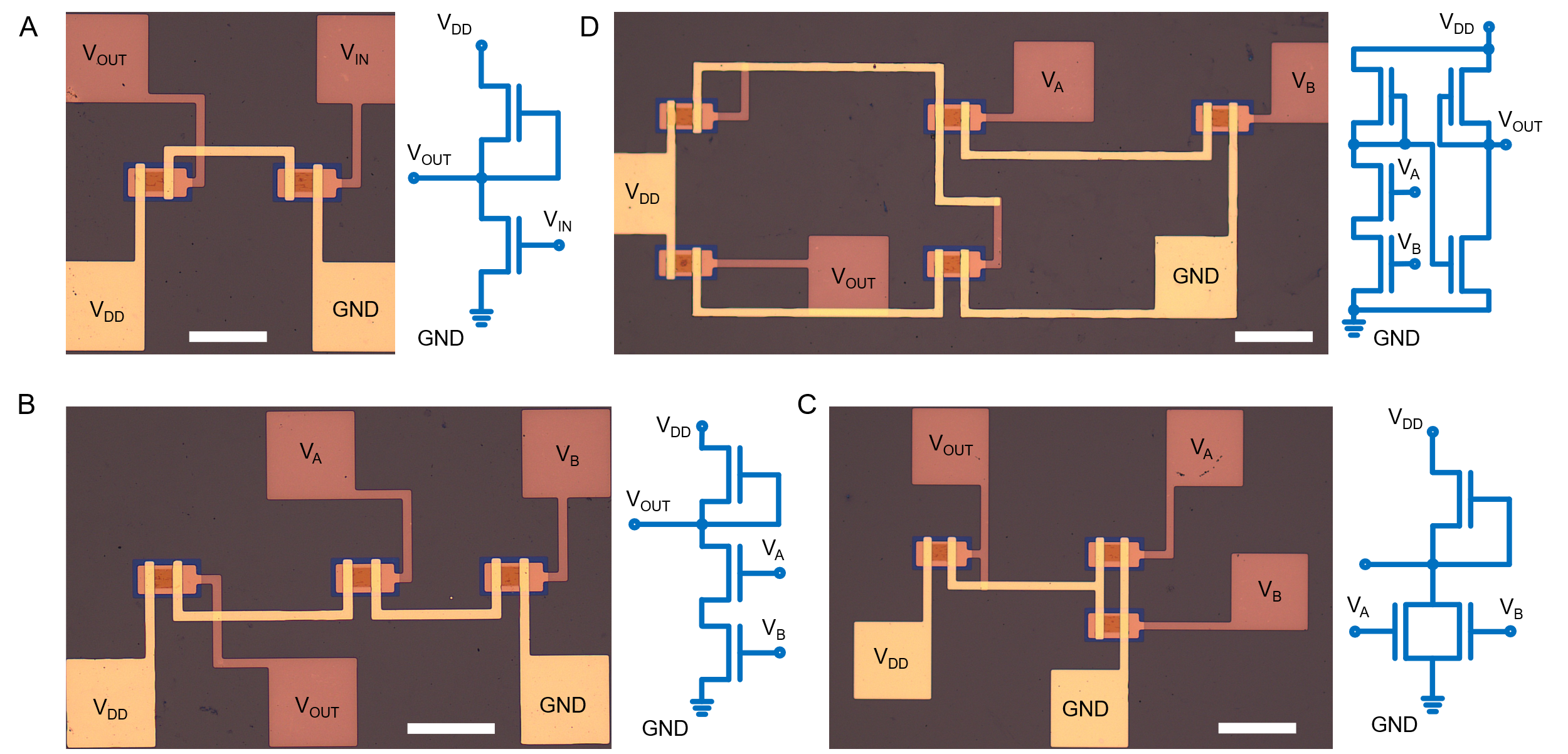
**

**Fig. S27.** (A to D) Optical images and schematics of inverter gate (A), NAND (B), NOR (C), and AND (D) logic circuits.


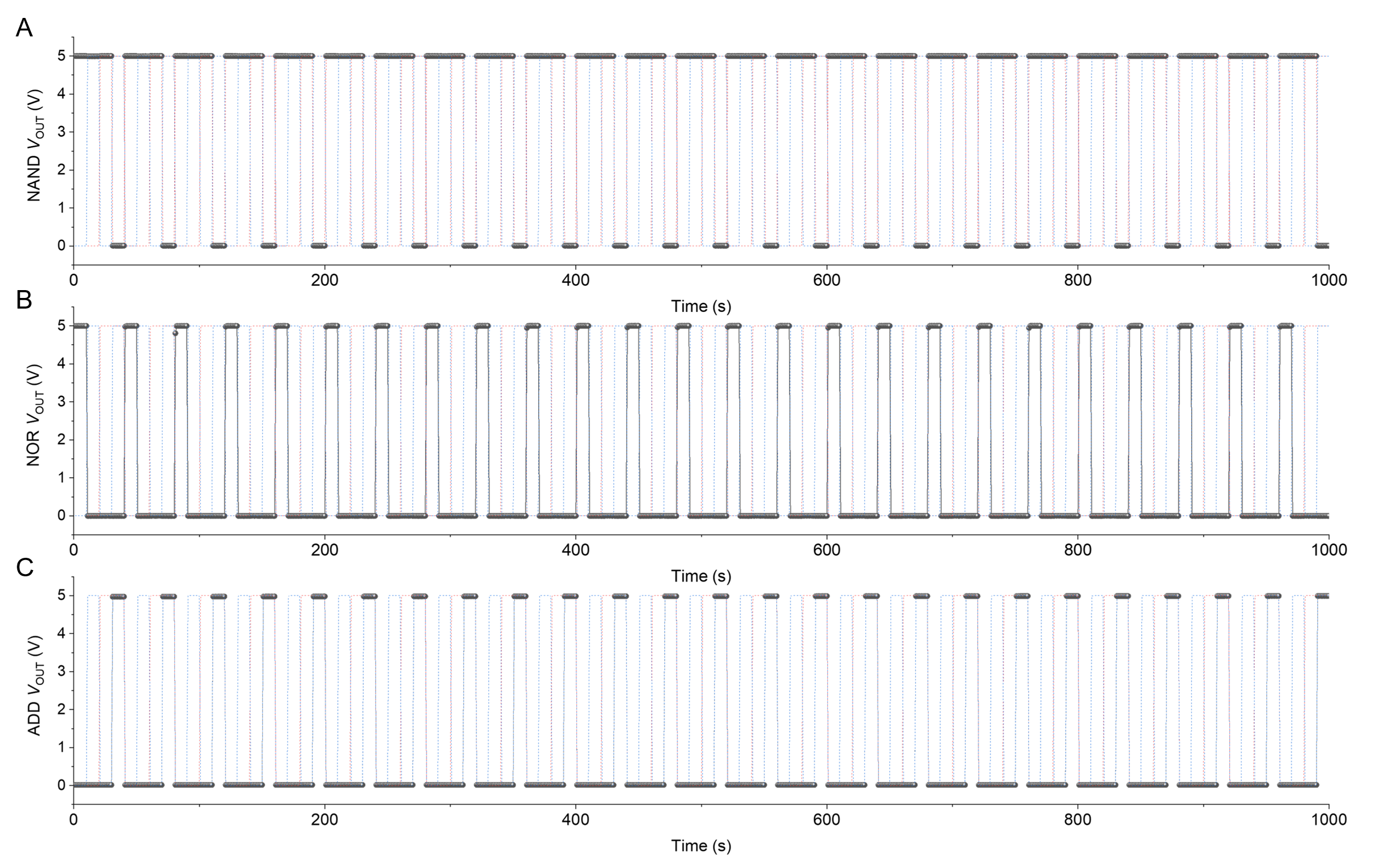


**Fig. S28.** (A to C) Waveforms of NAND (A), NOR (B), and AND (C) logic circuits.

# Supplementary Tables

**Table S1.** XPS Quantitative analysis results of the XPS spectra.

| **Name** | **Peak BE** | **FWHM eV** | **Atomic %** |
| --- | --- | --- | --- |
| **Mo 3d** | 229.36 | 0.85 | **35.84** |
| **S 2p** | 162.18 | 0.9 | **64.16** |

**Table S2.** Performance comparison of devices in references and this work.

| Materials | On-off Ratio | Mobility  (cm^2^ V^-1^ s^-1^) | *V*_TH_  (V) | SS  (mV/dec) | Gain  (*V*_DD_) | Power consumption  (*V*_DD_) | References |
| --- | --- | --- | --- | --- | --- | --- | --- |
| ME-CrOCl/MoS_2_ | 10^5^ | 425 | - | - | 16  (5 V) | 0.52 nW  (1 V) | 38 |
| CVD-MoS_2_ | 10^7^ | 70 | 0.96 | 75 | 2670  (4 V) | 412 nW  (1 V) | 1 |
| CVD-MoS_2_ | 10^6^ | 2.27 | -0.12 | 158 | 67  (2 V) | 0.02 nW  (0.25 V) | 33 |
| CVD-MoS_2_ | 10^7^ |  | -0.2 and 1.9 | 140 | 22.5  (3 V) | 1.2 nW  (2 V) | 34 |
| ME-MoS_2_ | 10^7^ | 52.6 | -3.0 – -1.9 | 100 | 17.8  (5 V) | 5 nW  (1 V) | 36 |
| MOCVD-MoS_2_ | 10^6^ | 40 | - | 65 – 75 | 4  (1 V) | - | 41 |
| MOCVD-MoS_2_ | 10^8^ | 9.1 | 5 | 370 | 7  (5 V) | - | 42 |
| CVD-MoS_2_ | 10^10^ | 59 | ~1 | 167 | 107  (4 V) | - | 40 |
| CVD-MoS_2_ | 10^4^ | 5.5 | - | - | 27  (5 V) | - | 39 |
| CVD-MoS_2_ | 10^8^ | 46 | 1.6 | 260 | 39  (3 V) | - | 35 |
| CVD-MoS_2_ | 10^7^ |  | 0.2 and 1.5 | 94 and 104 | 344  (4 V) | - | 32 |
| ME-MoS_2_ | 10^8^ | - | -4 – -2 | 76 | 36  (2 V) | - | 37 |
| **CVD-MoS_2_** | **10^8^** | **34.28** | **1.71** | **155.8** | **94**  **(4.5 V)** | **0.259 nW**  **(0.8 V)** | **This work** |

**Table S3.** Truth table of NAND, NOR, and AND gates.

| **Logic gate** | | | | |
| --- | --- | --- | --- | --- |
| ***V*_IN_ A (V)** | 0.00 (0) | 0.00 (0) | 5.00 (1) | 5.00 (1) |
| ***V*_OUT_ B (V)** | 0.00 (0) | 5.00 (1) | 0.00 (0) | 5.00 (1) |
| **NAND *V*_OUT_ (V)** | 5.00 (1) | 5.00 (1) | 5.00 (1) | 0.01 (0) |
| **NOR *V*_OUT_ (V)** | 4.93 (0) | 0.00 (0) | 0.00 (0) | 0.00 (0) |
| **AND *V*_OUT_ (V)** | 0.01 (0) | 0.01 (0) | 0.01 (0) | 4.99 (1) |
